# A Lethal Fungus Infects the Chinese White Wax Scale Insect and Causes Dramatic Changes in the Host Microbiota

**DOI:** 10.1038/s41598-018-23671-1

**Published:** 2018-03-28

**Authors:** Tao Sun, Xue-Qing Wang, Zun-Ling Zhao, Shu-Hui Yu, Pu Yang, Xiao-Ming Chen

**Affiliations:** 1 Research Institute of Resources Insects, Chinese Academy of Forestry, Key laboratory of Cultivating and Utilization of Resources Insects of State Forestry Administration, Kunming, 650224 China; 2 College of Agronomy, Kunming University, Kunming, 650214 China

## Abstract

The Chinese white wax scale insect (*Ericerus pela*) is an economically valuable species with an important role in wax production. Recently, in a greenhouse in Kunming, we identified a genus of fungus that infects and kills *E. pela* females. This study sought to perform the molecular detection of entomopathogens and analyze the changes in the host microbiota after entomopathogen infection. We used library construction, high-throughput sequencing and real-time quantitative polymerase chain reaction (RT-qPCR) to identify the fungi infecting adult *E. pela*, to understand the changes in the host organism, and to determine the distribution of the entomopathogens. *Cladosporium langeronii* and *C. sphaerospermum* were the main pathogenic species that infected the *E. pela* females, and they were most prevalent in the dorsal cuticle. *In vivo*, after infection, the proportion of *Cladosporium* clearly increased. The infection had little influence on the fungal community but had a strong influence on the bacterial community. After infection, *Arsenophonus* was dominant, and numerous bacterial genera disappeared. However, *Rickettsia*, instead of *Arsenophonus*, became dominant in the *Cladosporium-*infected individuals that had also been infected with *Rickettsia*. We identified the species that infected *E. pela* females and determined the influence of infection on the host microorganisms.

## Introduction

The scale insects are usually immobile on the host plants. They encounter adverse environmental conditions, predators, bacteria, and fungi. The wax secretion and other scale covers are produced by the scale insects to serve the protective function^1^. For the wax secretions, surface hydrophobic properties and antimicrobial activity come naturally. Therefore, the secretions produced by some scale insects have economic value and are used in industrial field^2^. The Chinese white wax scale insect, *Ericerus pela* Chavannes (Hemiptera: Coccoidae), is an economically valuable insect with an important role in wax production. The white wax secreted by the male second-instar nymph of *E. pela* is composed of natural esters and has wide applications in the medicine, food, chemical and other industries^3–9^. The white wax layer protects *E. pela* against attack by natural enemies and pathogens. It has evolved associations with microorganisms^2^. In a previous study, we analyzed the diversity of microorganisms in the white wax layer secreted by *E. pela*, and found the changes of bacterial and fungal communities in the wax layer with different thickness^2^. However, the studies of microorganisms in female secretion are lacking. Such studies are needed to better understand the influence of microorganisms in secretions on the host.

In traditional white wax production, the female and male *E. pela* are both reared to produce the eggs and white wax, respectively. In this process, the growth and development of females are influenced by several negative factors, which may result in decreased oviposition and hatchability. Because of the sedentary life on higher plant, females are susceptible to various pathogens. Phloem-feeding females excreted sugar-reach honeydew during adult stage. The excreted sugar increase sharply, and adhere to the immobile female adults with the development of females. The excreted sugar is prone to be contaminated by pathogens. Contamination of the excreted sugar is a serious threat to the survival of females.

**Figure 1:**
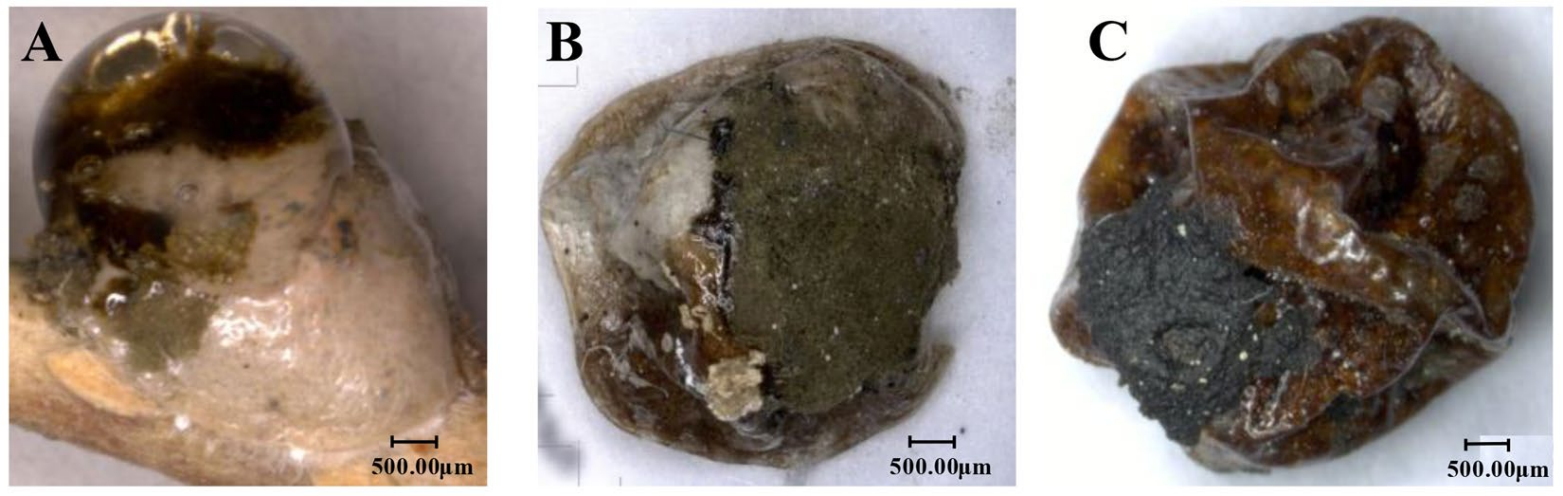
*E. pela* females at different infection stages. (**A**) Initial infection stage. The excreted sugar was contaminated and part of the sugar showed brown; however, the color of the female didn’t change. **(B)** Middle infection stage. The excreted sugar dries up and become olive green or more dark, the color of the female become brown. **(C)** Last infection stage. The female shrivel and die.

Over the past two years, we found that many females were infected with a green microorganism and died in the greenhouse in Kunming. Through detailed observations of the infected insects, we found that the microorganism parasitized initially on the sugar excreted from the female adult vent during the oviposition period, on the cuticle of *E. pela* (Fig. 1A). After the excreted sugar dries up, the microorganism surrounds the cuticle (Fig. 1B). Ultimately, the infected insects shrivel and die (Fig. 1C). We also observed that the color of the microorganism changes from olive green to dark green during the drying of the excreted sugar and the degeneration of the female *E. pela* in the infection process. We observed that the pathogen had spread and infected many *E. pela* in the greenhouse within one month.

Because the microorganism that we observed harmed the females of *E. pela*, we inferred that it might be a new type of pathogenic microorganism that kills the host by constantly expanding the regions that it parasitizes from the outside in. In this study, we detected the major microorganism parasitizing on the sugar secreted by female *E. pela* by 16S and ITS (internal transcribed spacer) library construction and sequencing; then, we used the relative RT-qPCR technique to identify the infected tissues. The microbial communities of the infected females were analyzed by high-throughput sequencing. This study lays a foundation for further investigations of the microorganisms parasitizing *E. pela* and should provide a scientific basis for the management of microorganism infection during *E. pela* breeding. In addition, the study may be helpful in the management of other scale insects through use of this microorganism as a biological control.

## Results

### Library analysis and sequencing

#### The 16S library

No 16S rDNA genes were amplified from bacterial DNA. The results demonstrated that there were no bacteria in the infected samples of excreted sugar (Figure S1).

#### The ITS library

The titers of the three ITS libraries were 5.39 × 10^6^, 1.79 × 10^6^ and 2.26 × 10^6^, and the recombination rates were 95.91%, 97.46% and 94.46%. Therefore, the three libraries were suitable for identifying the fungi found on the excreted sugar and the outer cuticle of the females. The BLAST results showed that the 30 sequences were from 6 species of fungi: *C. langeronii* (10 sequences), *C. sphaerospermum* (16 sequences), *C. cladosporioides* (1 sequence), *C. sp. MPI.04* (1 sequence), *C. sp. NJP05* (1 sequence), and *Cryptococcus sp. SJ8L03* (2 sequence).

### The results of 16S and ITS MiSeq high-throughput sequencing

#### Statistical summaries

The read numbers and OTUs of bacteria and fungi in the control checks (CK) and the infection groups (IN) are shown in Table 1. The rarefaction curve and Shannon-Wiener curve showed the number of read samples reached represented saturated conditions (Figure S2).

**Table 1:** The numbers of reads and OTUs of bacteria and fungi in CK and IN.

		CK1	CK2	CK3	Average	IN1	IN2	IN3	Average
Bacteria	Reads	22,808	26,085	27,776		29,908	38,179	20,662	
	OTUs	525	523	62	369	25	62	98	61
Fungi	Reads	28,757	29,075	34,833		29,958	29,502	24,304	
	OTUs	39	53	60	48	82	53	60	67

The average number of bacterial OTUs of IN was 308 less than in CK. The average number of fungal OTUs of IN was 19 more than in CK (Table 1).

#### Bacterial diversity and community structure

After the female *E. pela* were infected, the bacterial community richness was clearly reduced, as shown by the significant reduction of the Chao index and Ace index in IN compared with CK. The bacterial community diversity was also markedly reduced, as shown by the increasing Simpson index and significant reduction of the Shannon index in IN compared with CK. The Simpson index was 0.2736 in CK and 0.8648 in IN, which indicated the proportion of dominant species was not high in CK but was high in IN (Table 2).

**Table 2:** The diversity of bacteria and fungi in CK and IN.

Microorganism	Sample	Ace	Chao	Shannon	Simpson
Bacteria	CK	386.67	392.63	3.83	0.2736
	IN	82.85	73.88	0.44	0.8648
Fungi	CK	68.65	61.02	1.50	0.3622
	IN	74.39	77.55	1.77	0.2676

In total, 25 phyla, and 253 genera were identified. The bacterial community in CK was dominated by the Proteobacteria phyla (68.15%), followed by Firmicutes (7.18%). It was clearly different in IN. The proportion of Proteobacteria was dominant (93.86%) after infection. However, the proportions of six phyla were reduced significantly after infection, and ten other phyla disappeared after infection (Table S1).

A total of 236 genera were identified in CK, whereas only 89 genera were identified in IN. A total of 164 genera disappeared after infection, including *Desulfobulbus*, *Nitrosococcus*, and *Sh765B-TzT-29*. However, 17 genera appeared after infection, the main genus of which was *Arsenophonus* (Table S1).

One CK sample (CK3) and one IN sample (IN3) was found to have been infected with *Rickettsia*, which accounted for the vast majority of bacteria (90.22% in CK and 88.06% in IN). To avoid the influence of the *Rickettsia* bacteria on the analysis, the IN3 and CK3 pair was selected for comparison alone.

In the other two CK groups (CK1 and CK2), we did not find *Rickettsia* was dominant, as expected, but after infection (IN1 and IN2), *Arsenophonus* was dominant and several bacterial genera disappeared. The details are as follows:

In the two CK groups, a total of 226 genera were identified, with the highest proportion represented by *Rhodospirillaceae_uncultured* (5.66%). In the two IN groups, the community was entirely different. There were 181 bacterium genera that disappeared after infection, and only 57 genera were identified, of which *Arsenophonus* was dominant (95.20%), followed by *Bacillus* (1.64%), *Paenibacillus* (1.12%), and others (2.04%).

However, findings in IN3 (has been infected with *Rickettsia*), were different. The proportion of *Arsenophonus* was only 0.37% in IN3. A total of 69 genera were identified, and *Rickettsia* was dominant (88.06%), followed by *Bacillus* (4.12%) and *Paenibacillus* (2.27%). In CK3 (has been infected with *Rickettsia*), only 51 genera were identified, of which *Rickettsia* was dominant (90.22%), followed by *Bacillus* (4.10%) and *Paenibacillus* (2.58%). Aside from the reduced proportion of *Rickettsia* in IN3, we did not find additional differences between IN3 and CK3.

#### Fungal diversity and community structure

The fungal community richness increased after the female *E. pela* were infected, as shown by the increased Chao index and Ace index after infection (IN vs. CK). The fungal community diversity also increased, as shown by the reduction of the Simpson index and the increased Shannon index (Table 2).

A total of 4 phyla, and 69 genera were identified. At the phylum level, we did not find significant differences between CK and IN. However, Zygomycota was absent in CK but present in IN (0.02%). The phylum Ascomycota (93.92%) was dominant in CK. This composition was similar in IN (Table S1).

At the genus level, in CK, *Cladosporium* was dominant (64.09%), followed by *Ophiocordycipitaceae* (24.31%), *Basidiomycota* (5.74%) and *Davidiellaceae* (2.78%). However, after infection, the proportions of *Cladosporium* increased to 75.68%, followed by *Ophiocordycipitaceae* (11.39%), *Basidiomycota* (5.54%) and *Cryptococcus* (2.78%). In addition, *Davidiellaceae* (2.78%) was found in CK but was absent in IN, and *Cryptococcus* (2.78%) was found in IN but was absent in CK (Table S1).

**Figure 2:**
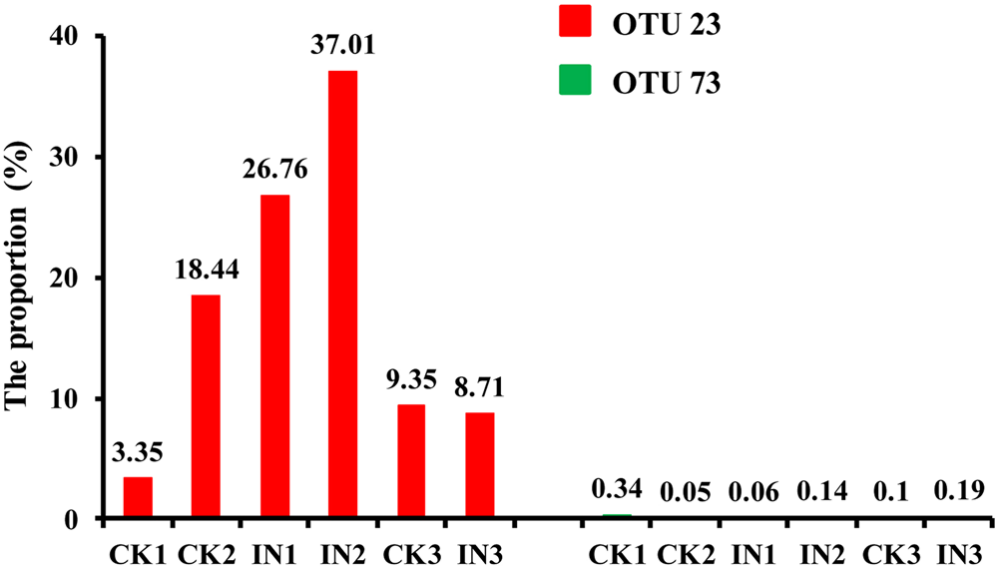
The proportion of OTU 23 and OTU 73 in the three control checks (CK1, CK2, and CK3) and three infection groups (IN1, IN2, and IN3). The OUT 23 was shown in red color, and the OUT 73 was shown in green color.

Because *Cladosporium* was identified in both the excreted sugar and normal female individuals, we further analyzed the sequence of *Cladosporium* in detail. The sequences of *C. langeronii* and *C. sphaerospermum* from the ITS library were aligned with the sequences of OTUs, which were classified into the *Cladosporium* genus. The ITS I fragment of *C. langeronii* had 100% similarity with OTU 73, and the ITS I fragment of *C. sphaerospermum* had 100% similarity with OTU 23 (Figure S3). The proportion of OTU 23 and OTU 73 in each sample is shown in Fig. 2. However, the proportion of OTU 73 was very low in each sample (Fig. 2).

### *C. langeronii and C. sphaerospermum* distribution in the female body after infection

From the results of relative RT-qPCR, we found that *C. langeronii* and *C. sphaerospermum* were expressed in all the tissues we dissected. However, in different individuals, different tissues had different expression levels. We found that the expression levels of the two genes were highest in the dorsal cuticle, followed by fat body. The lowest expression level was in the ovaries (Figure S4).

## Discussion

In this study, we identified the fungi that infected *E. pela*. ITS library analysis showed that *Cladosporium* was the major genus that infected *E. pela*. There were two species: *C. langeronii* and *C. sphaerospermum*. *Cladosporium* belongs to the anamorphic fungi, the Cladosporiaceae. *Cladosporium* has a worldwide distribution and often parasitizes plants, humans and animals, causing sickness. The spore can be allergenic and can cause subcutaneous phaeohyphomycosis^10,11^. Many researchers have found that *Cladosporium* is an effective entomopathogenic fungus against many insect pests^12,13^. However, *Cladosporium* had not previously been found to infect scale insects. This finding might provide a new means for controlling scale insect pests.

*C. langeronii* and *C. sphaerospermum* showed the highest expression levels in the dorsal cuticle of the adults, a result consistent with the infection process from outside to inside. However, *Cladosporium* was also found in normal female individuals. It is thought that the pathogen may be suppressed by the insect’s resistance measures, such as cellular immunity and cellular immunity, antimicrobial peptides (AMPs) and symbiotic bacteria^14^. It can be inferred that, the low content of *C. langeronii* and *C. sphaerospermum* cause no harm to the normal individuals (Figure S5). *C. langeronii* and *C. sphaerospermum* in the normal female individuals will dormant forever until they meet favorable conditions for expansion. It is similar to the *Wolbachia*, which is a symbiont normally, can cause early death of *Drosophila*^15^. We also identified *Cladosporium* in the wax layer secreted by male *E. pela*. Their content decreased from 17.01 to 0.49% with the wax layer thickening^2^. It seemed that *Cladosporium* in the wax layer has no influence on the survival of the male *E. pela*. More specific primers that facilitate more accurate identification will be needed to analyze the distribution of *Cladosporium* in the female and male *E. pela*^16,17^.

After infection, the proportion of OTU 23 increased significantly but the proportion of OTU 73 did not substantially change. The variation in OTU 23 proportion among the three infection samples maybe results from the individual differences during female defense to fungal infection.

On the basis of the observation of infected female *E. pela*, most of the dead individuals were infected from the outside in. Hence, we speculate that one of the female *E. pela* died may also because of fungal mechanical force or fungal toxins. In a study of *Beauveria bassiana*, researchers have also found that the fungus can damage the host *Carposina sasakii* through mechanical force^18^. Other researchers have found that fungal toxins can lead to host death^19,20^. Hence, the death of *E. pela* females may be caused by these factors.

**Figure 3:**
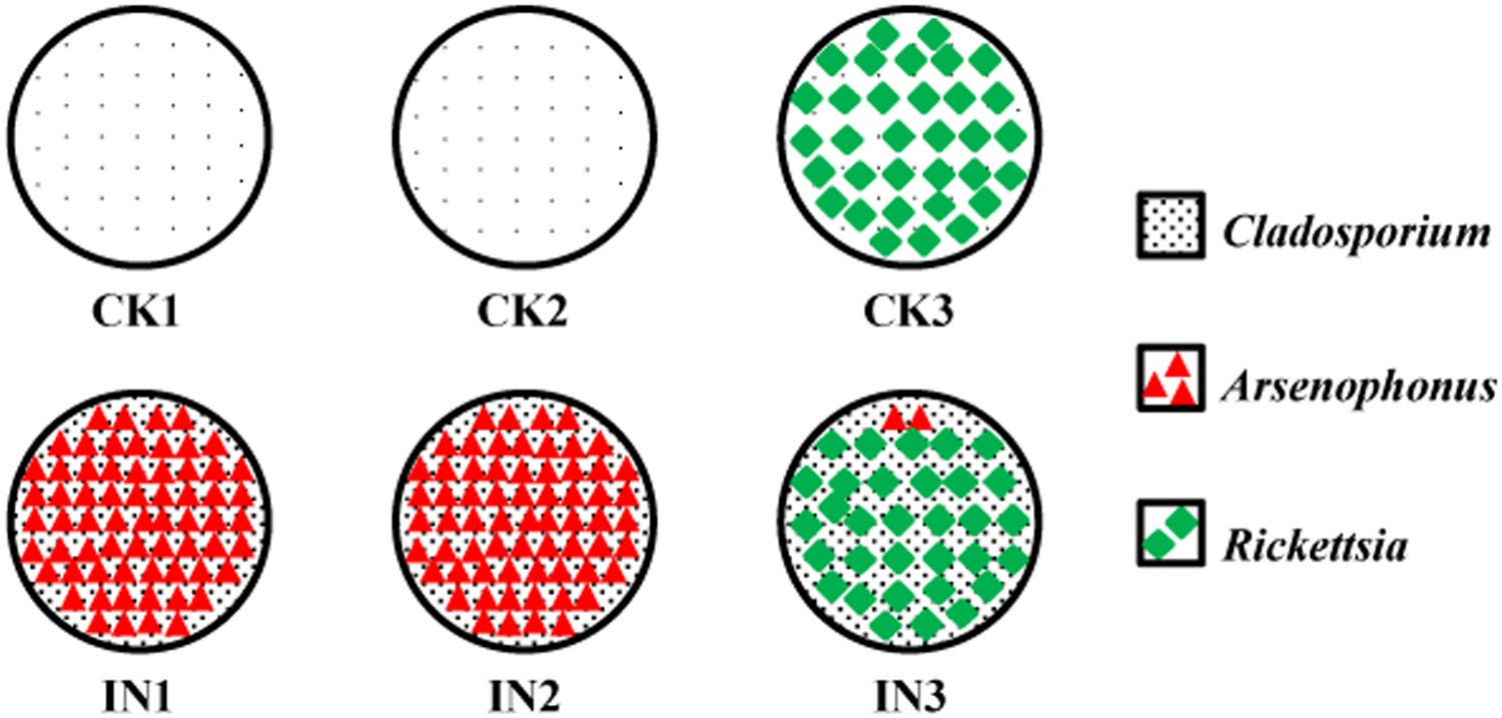
The sketch map that showed the distribution of *Cladosporium*, *Arsenophonus* and *Rickettsia* in the six samples (CK1, CK2, CK3, IN1, IN2, and IN3). The sketch map showed that, *Arsenophonus* increased after infection (IN1, IN2); however, if the individuals have been infected with *Rickettsia*, *Arsenophonus* colonies may be inhibited by *Rickettsia*.

**Figure 4:**
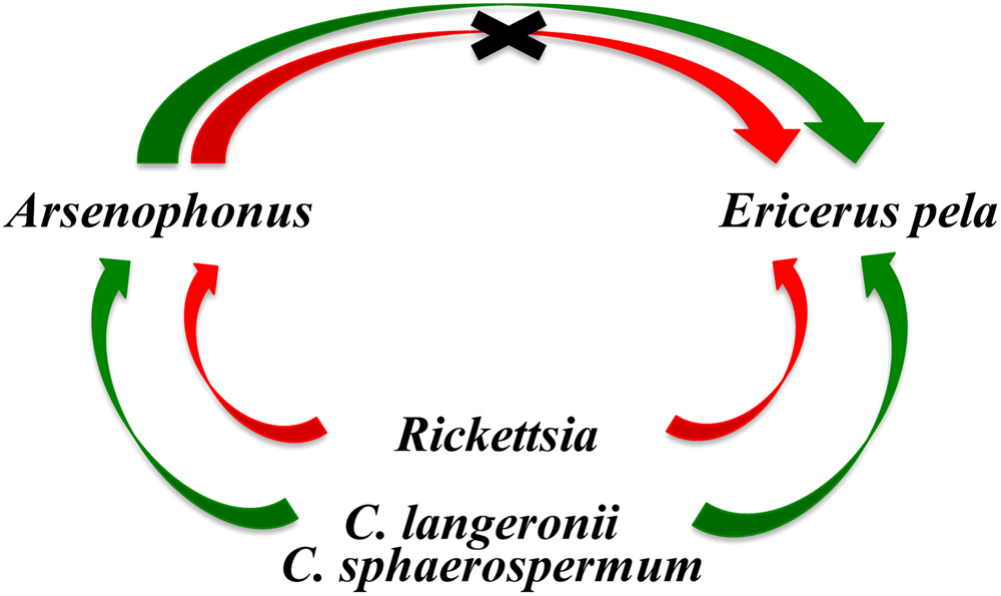
The relationship among microorganisms in *E. pela* females and the interaction between females and endosymbiont. The figure showed that, *C. langeronii* and *C. sphaerospermum* infection may promote *Arsenophonus* colonies in *E. pela*; however, *Rickettsia* infection can shield against this effects.

*Arsenophonus* is considered to be a secondary endosymbiont in *E. pela*^21^. Researchers have found that symbiotic bacteria are helpful in protecting the host against pathogens and play an important role in host physiology and metabolism^22,23^. In this study, we found that after *C. langeronii* and *C. sphaerospermum* infection, female *E. pela* were more susceptible to infection with *Arsenophonus* (Fig. 3). *C. langeronii* and *C. sphaerospermum* infection may promote *Arsenophonus* transmission horizontally or infection in *E. pela* females (Fig. 4). A similar finding has also been reported in *Glycaspis brimblecombei* larvae^24^ and *Ixodiphagus hookeri*^25^. Thus, we speculate that *Arsenophonus* infection might be a form of countermeasure against *C. langeronii* and *C. sphaerospermum*.

Interestingly, we found that *Arsenophonus* colonies may be inhibited by *Rickettsia*. The proportion of *Arsenophonus* was only 0.37% in IN3 (Fig. 3). Thus, after the host is infected with *Rickettsia*, the promotion effect of *C. langeronii* and *C. sphaerospermum* on *Arsenophonus* infection is negligible. In addition, *Rickettsia* infection can shield against the effects of *C. langeronii* and *C. sphaerospermum* infection of the host (Fig. 4). *Rickettsia* is also considered to be a secondary endosymbiont in *E. pela*^26^. Many researchers have found that *Rickettsia* can aid in preventing pathogen invasion^27–29^.

Secondary endosymbionts have been shown to help protect the host against parasitoids and fungal entomopathogens and to potentially influence host physiology, evolution and distribution^24,30,31^. In this study, we identified the lethal fungus infects *E. pela* and inferred the microorganism interactions. It is useful to identify the insect-endosymbiont interactions and may provide a reference for future studies investigating entomopathogens in *E. pela*.

## Methods

### Materials

*Insects*. *E. pela* females were collected from Chinese privet (*Ligustrum sinense* Lour) in the greenhouse of the Research Institute of Resource Insects in Kunming (102.73°E, 25.04°N).

Infected samples of excreted sugar used for microorganism isolation were collected from the infected females’ bodies during the middle infection stage (Fig. 1B) because the biomass of microorganisms from the middle stage is higher than that in the initial stage, and the females in this stage of infection are still alive, as shown in Fig. 1.

### Library construction of 16S and ITS

#### Isolation of microorganism genomic DNA

The microorganism samples (which were dark green and surrounded by the dry excreted sugar, as shown in Fig. 1) were extracted from six different females, three of which were used to isolate fungal DNA (F1, F2, F3) and the other three of which were used to isolate bacterial DNA (B1, B2, B3). Then, the bacterial DNA of the three microorganism samples was isolated separately by using a Bacterial DNA Miniprep Kit (Omega, Atlanta, GA, USA), and the fungal DNA of the other three microorganism samples was isolated separately using the Fungal DNA Miniprep Kit (Omega, Atlanta, GA, USA).

#### 16S and ITS amplification, cloning, and library construction

Three 16S rDNA genes were amplified using the bacterial DNA as a template and the universal primers (27F and 1492R)^32^. However, when the production was separated on a 1% agarose gel, no amplification bands were visible (Figure S1).

The ITS genes containing the ITS I and ITS II regions were amplified (ITS1 and ITS4; the primer sequences were according to Bokulich *et al*., 2013)^33^ by using the fungal DNA as a template. Three biological repeats were used. Then, three ITS rDNA libraries were constructed.

#### Library analysis and sequencing

After blue-white selection and positive clone identification by using bacterial PCR, the colony-forming units (cfu) and recombination rate were calculated according to Sambrook *et al*., 2014^34^.

Ten positive recombinant monoclones were selected at random from every library and then sequenced by BioSune (Kunming, China). Then, the sequences were annotated with the NCBI basic local alignment search tool (BLAST) (https://blast.ncbi.nlm.nih.gov/Blast.cgi) under the default parameter values, and the results were selected on the basis of identity thresholds ≥95%.

### *E. pela* female microbial diversity analysis based on 16S and ITS MiSeq high-throughput sequencing

#### Isolation of the genomic DNA of E. pela females

A total of six females were collected for high-throughput analysis: three individuals were infected with the microorganism (IN1, IN2, IN3), and the other three individuals were control checks (CK1, CK2, CK3). The six samples were crushed into powder separately, as previously described for DNA isolation, with a DNeasy Blood and Tissue Kit (QIAGEN, Hilton, Germany). Then, the DNA was stored at −20 °C for MiSeq sequencing of 16S and ITS.

#### MiSeq sequencing of 16S and ITS

The genomic DNA from the six females was used separately as a template to amplify 16S rRNA genes or the ITS regions by using primers (338F and 806R for 16S rRNA genes; ITS1F and ITS2R for ITS regions; the primer sequences were according to Xu *et al*. and Bokulich *et al*.)^21,33^ with different barcodes. The 100 μL PCR reaction system contained 500 ng DNA template, 0.2 mM primer, 0.2 mM dNTP mix and 2.5 U of Q5^®^ High-Fidelity DNA Polymerase (NEB, Ipswich, MA, USA). The PCR conditions were 94 °C for 5 minutes, then 25 cycles at 94 °C for 30 seconds, 55 °C for 30 seconds, and 72 °C for 1 minute. The PCR was purified with a Gel Extraction Kit (Thermo, Waltham, MA, USA) and then stored at −20 °C used.

The products were detected and quantified with QuantiFluor^TM^-ST (Promega, Madison, WI, USA). The loading quantities were calculated to determine the sequencing requirements.

The 16S and ITS MiSeq PE (paired-end) libraries were constructed as follows: First, the products were linked with “Y”-type adapters. Then, the filtered double-stranded DNA segments were enriched via PCR amplification. The products were denatured by sodium hydroxide to produce single-stranded DNA segments. Finally, the single-stranded DNA segments were sequenced by using Illumina MiSeq (Illumina, San Diego, California, USA).

### Data analysis

According to the overlap nexus, the PE reads were assembled using FLASH software (Version 3+). The quality of reads and the effect of assembly were simultaneously detected. The detected reads were quality filtered to remove the adapters, unreplicated single sequences and chimeric reads, and effective reads were obtained. Because the six samples resulted in different numbers of reads, 12,476 reads in bacteria 16S and 24,227 reads in fungi ITS were selected at random for each sample to provide the same amount of data for further analysis.

Then, the reads were analyzed by using Usearch software (Version 7.1 http://drive5.com/uparse/) to generate operational taxonomic units (OTUs) at the 97% identity level. On the basis of the OTUs data, the taxonomy and the alpha-diversity were analyzed and calculated.

In the taxonomy analysis, the OTUs were analyzed by using the RDP Classifier (Version 2.2 http://sourceforge.net/projects/rdp-classifier/), and the community composition was analyzed at different taxonomic levels, including domain, kingdom, phylum, class, order, family, genus and species.

In the alpha-diversity analysis, the Chao, Ace, Shannon, Simpson and Coverage indexes were calculated by using Mothur software (Version v.1.30.1 http://www.mothur.org/wiki/Schloss_SOP#Alpha_diversity) on the basis of the OTUs.

The rarefaction curve and Shannon-Wiener curve were drawn by using Mothur software (Version v.1.30.1 http://www.mothur.org/wiki) and R software.

### Relative RT-qPCR

#### DNA isolation in different tissues

The fat body, enteron, cuticle, and ovary were dissected from 3 infected females by using sterile microforceps under an anatomical lens. Each tissue was separately washed for 2 minutes in sterile water, and the tissue was immersed in lysis buffer containing proteinase K (Roche, Mannheim, Germany) and then incubated for 30 minutes in hot water. After a brief centrifugation, the same volume of sterile water was added to the supernatant, mixed gently, and stored at −20 °C for relative RT-qPCR analysis. Each tissue was subjected to 3 replications and used for DNA isolation.

#### Relative RT-qPCR

According to the results from the ITS library sequencing, specific primers (Figure S6) for *C. langeronii* and *C. sphaerospermum* (the fungus infects *E. pela* identified by the method stated above) were designed. The 10 µL PCR solution with SsoFast EvaGreen Supermix (Bio-Rad, Hercules, CA, USA) was used. The RT-PCR reactions were performed with a CFX96^TM^ real-time system (Bio-Rad, Hercules, CA, USA). The cycling conditions were 98 °C for 3 minutes, then 39 cycles at 98 °C for 10 seconds and 62 °C for 20 seconds. The solubility curves were generated by increasing the annealing temperature at 5 °C/5 seconds from 65 °C to 95 °C. The succinate dehydrogenase subunit A (SdhA) gene and myosin (Myo) gene were selected as two reference genes for relative RT-qPCR. Each PCR run was repeated 3 times.

The results were analyzed using the LSD (least significant difference) method at P < 0.01 level in the Data Processing System (DPS) software^35^.

## Electronic supplementary material

Supplementary Information
