# Supplementary material for: A Lethal Fungus Infects the Chinese White Wax Scale Insect and Causes Dramatic Changes in the Host Microbiota
Source: Sci Rep. 2018 Mar 28;8:5324. doi: 10.1038/s41598-018-23671-1 (PMC5871785; doi:10.1038/s41598-018-23671-1)
Supplement: Supplementary file 1 — Supplementary Information [file 41598_2018_23671_MOESM1_ESM.doc]

**A Lethal Fungus Infects the Chinese White Wax Scale Insect and Causes Dramatic Changes in the Host Microbiota**

Tao Sun^#1^, Xue-Qing Wang^#1^, Zun-Ling Zhao^#1^, Shu-Hui Yu^2^, Pu Yang^1^* & Xiao-Ming Chen^1^*

^1^ Research Institute of Resources Insects, Chinese Academy of Forestry, Key laboratory of Cultivating and Utilization of Resources Insects of State Forestry Administration, Kunming, 650224, China

^2^ College of Agronomy, Kunming University, Kunming, 650214, China.

# These authors contributed equally to this work.

Correspondence and requests for materials should be addressed to P. Y. (E-mail: [zjuyangpu@aliyun.com](mailto:zjuyangpu@aliyun.com))

**Table Supplementary material:**

**Table S1. The distribution of bacteria and fungi in the infected females and normal females.**

| **Bacteria** | **Taxon** | **CK1** | **CK2** | **CK3** | **IN1** | **IN2** | **IN3** | **Fungi** | **Taxon** | **CK1** | **CK2** | **CK3** | **IN1** | **IN2** | **IN3** |
| --- | --- | --- | --- | --- | --- | --- | --- | --- | --- | --- | --- | --- | --- | --- | --- |
| **Phylum (percents)** | Acetothermia | 0.0002 | 8.02E-05 | 0.0000 | 0.0000 | 0.0000 | 0.0000 | **Phylum (percents)** | Ascomycota | 0.9477 | 0.9439 | 0.9260 | 0.8877 | 0.9702 | 0.8587 |
|  | Acidobacteria | 0.0478 | 0.0468 | 0.0000 | 0.0000 | 0.0000 | 0.0002 |  | Basidiomycota | 0.0511 | 0.0558 | 0.0737 | 0.1115 | 0.0297 | 0.1412 |
|  | Actinobacteria | 0.0571 | 0.0620 | 0.0027 | 0.0002 | 0.0029 | 0.0036 |  | Fungi_unclassified | 0.0012 | 0.0003 | 0.0003 | 0.0003 | 0.0001 | 0.0001 |
|  | Bacteria_unclassified | 0.0015 | 0.0017 | 0.0000 | 0.0000 | 0.0000 | 0.0000 |  | Zygomycota | 0.0000 | 0.0000 | 0.0000 | 0.0005 | 0.0000 | 0.0000 |
|  | Bacteroidetes | 0.0749 | 0.0697 | 0.0034 | 0.0008 | 0.0028 | 0.0064 | **Genus (percents)** | Acaromyces | 0.0033 | 0.0017 | 0.0010 | 0.0210 | 0.0072 | 0.0007 |
|  | Chlamydiae | 0.0000 | 0.0000 | 0.0000 | 0.0000 | 8.02E-05 | 0.0000 |  | Acremonium | 0.0007 | 0.0006 | 0.0002 | 0.0002 | 0.0038 | 0.0019 |
|  | Chlorobi | 0.0065 | 0.0067 | 0.0000 | 8.02E-05 | 8.02E-05 | 8.02E-05 |  | Alternaria | 4.13E-05 | 0.0007 | 0.0001 | 0.0005 | 0.0003 | 0.0002 |
|  | Chloroflexi | 0.0273 | 0.0253 | 0.0006 | 8.02E-05 | 0.0002 | 0.0014 |  | Amphisphaeriaceae_unclassified | 0.0000 | 8.26E-05 | 0.0000 | 0.0000 | 0.0000 | 0.0000 |
|  | Cyanobacteria | 0.0038 | 0.0047 | 0.0003 | 0.0007 | 0.0031 | 0.0035 |  | Ascomycota_unclassified | 8.26E-05 | 4.13E-05 | 0.0000 | 0.0022 | 0.0008 | 0.0016 |
|  | Deferribacteres | 0.0039 | 0.0038 | 0.0000 | 0.0000 | 0.0000 | 0.0000 |  | Aspergillus | 0.0000 | 4.13E-05 | 4.13E-05 | 4.13E-05 | 4.13E-05 | 0.0002 |
|  | Fibrobacteres | 0.0004 | 0.0002 | 0.0000 | 0.0000 | 0.0000 | 0.0000 |  | Aureobasidium | 0.0000 | 0.0000 | 0.0011 | 0.0005 | 4.13E-05 | 0.0009 |
|  | Firmicutes | 0.0664 | 0.0709 | 0.0782 | 0.0008 | 0.0666 | 0.0835 |  | Basidiomycota_unclassified | 0.0478 | 0.0534 | 0.0712 | 0.0501 | 0.0163 | 0.0998 |
|  | Fusobacteria | 0.0030 | 0.0025 | 0.0000 | 0.0000 | 0.0000 | 0.0004 |  | Blumeria | 0.0000 | 0.0000 | 0.0000 | 0.0000 | 4.13E-05 | 0.0000 |
|  | Gemmatimonadetes | 0.0192 | 0.0190 | 0.0000 | 0.0000 | 0.0002 | 8.02E-05 |  | Botryosphaeriaceae_unclassified | 0.0000 | 0.0000 | 0.0000 | 0.0000 | 0.0000 | 8.26E-05 |
|  | JL-ETNP-Z39 | 0.0005 | 0.0006 | 0.0000 | 0.0000 | 0.0000 | 0.0000 |  | Capnodiales_unclassified | 0.0001 | 0.0002 | 0.0001 | 0.0007 | 8.26E-05 | 4.13E-05 |
|  | Latescibacteria | 0.0031 | 0.0019 | 0.0000 | 0.0000 | 0.0000 | 0.0000 |  | Chaetothyriaceae_unidentified | 0.0000 | 0.0000 | 0.0000 | 0.0000 | 0.0000 | 0.0006 |
|  | Lentisphaerae | 0.0002 | 8.02E-05 | 0.0000 | 0.0000 | 0.0000 | 0.0000 |  | Chalastospora | 0.0000 | 0.0000 | 0.0000 | 0.0001 | 0.0000 | 0.0000 |
|  | Nitrospirae | 0.0701 | 0.0701 | 0.0006 | 0.0000 | 0.0003 | 0.0002 |  | Cladosporium | 0.8706 | 0.5541 | 0.4979 | 0.6603 | 0.8726 | 0.7374 |
|  | Parcubacteria | 0.0003 | 0.0002 | 0.0000 | 0.0000 | 0.0000 | 0.0003 |  | Coniothyrium | 0.0000 | 0.0000 | 8.26E-05 | 0.0000 | 4.13E-05 | 0.0000 |
|  | Planctomycetes | 0.0357 | 0.0356 | 0.0000 | 0.0000 | 0.0000 | 0.0000 |  | Cryptococcus | 4.13E-05 | 0.0002 | 0.0007 | 0.0379 | 0.0059 | 0.0397 |
|  | Proteobacteria | 0.5668 | 0.5660 | 0.9117 | 0.9974 | 0.9210 | 0.8973 |  | Cryptovalsa | 0.0000 | 0.0000 | 0.0000 | 4.13E-05 | 0.0000 | 0.0000 |
|  | SHA-109 | 0.0002 | 0.0002 | 0.0000 | 0.0000 | 0.0000 | 0.0000 |  | Curvularia | 0.0000 | 4.13E-05 | 0.0000 | 4.13E-05 | 0.0000 | 0.0000 |
|  | Spirochaetae | 0.0100 | 0.0107 | 0.0000 | 0.0000 | 0.0000 | 0.0002 |  | Cystofilobasidium | 0.0000 | 4.13E-05 | 0.0000 | 0.0001 | 0.0000 | 0.0000 |
|  | TM6 | 8.02E-05 | 8.02E-05 | 0.0000 | 0.0000 | 0.0000 | 0.0000 |  | Davidiella | 4.13E-05 | 8.26E-05 | 0.0000 | 0.0005 | 0.0000 | 0.0001 |
|  | Verrucomicrobia | 0.0009 | 0.0011 | 0.0026 | 0.0000 | 0.0026 | 0.0028 |  | Davidiellaceae_unclassified | 0.0002 | 0.0002 | 0.0829 | 0.0039 | 0.0002 | 0.0031 |
| **Genus (percents)** | 43F-1404R_norank | 0.0010 | 0.0006 | 0.0000 | 0.0000 | 0.0000 | 0.0000 |  | Devriesia | 0.0000 | 8.26E-05 | 0.0002 | 4.13E-05 | 0.0020 | 0.0000 |
|  | 480-2_norank | 0.0009 | 0.0008 | 0.0000 | 0.0000 | 0.0000 | 0.0000 |  | Diatrypella | 4.13E-05 | 0.0000 | 4.13E-05 | 0.0000 | 0.0001 | 0.0002 |
|  | A0839_norank | 0.0000 | 0.0000 | 0.0000 | 0.0000 | 0.0002 | 8.02E-05 |  | Epicoccum | 4.13E-05 | 0.0002 | 0.0003 | 0.0000 | 0.0000 | 0.0001 |
|  | AEGEAN-245_norank | 0.0002 | 8.02E-05 | 0.0000 | 0.0000 | 0.0000 | 0.0000 |  | Erysiphe | 0.0000 | 0.0000 | 0.0000 | 4.13E-05 | 0.0000 | 0.0000 |
|  | Acidimicrobiaceae_unclassified | 0.0000 | 0.0000 | 0.0000 | 0.0000 | 0.0000 | 0.0003 |  | Erythrobasidium | 0.0000 | 0.0000 | 0.0000 | 0.0002 | 0.0000 | 0.0000 |
|  | Acidimicrobiales_uncultured | 0.0033 | 0.0034 | 0.0000 | 0.0000 | 0.0000 | 0.0000 |  | Exobasidium | 0.0000 | 0.0001 | 0.0007 | 0.0002 | 0.0002 | 0.0003 |
|  | Acidobacteria_norank | 0.0172 | 0.0174 | 0.0000 | 0.0000 | 0.0000 | 8.02E-05 |  | Fungi_unclassified | 0.0012 | 0.0003 | 0.0003 | 0.0003 | 0.0001 | 0.0001 |
|  | Acinetobacter | 0.0002 | 0.0000 | 0.0002 | 0.0002 | 0.0003 | 0.0002 |  | Golovinomyces | 0.0000 | 0.0000 | 0.0001 | 0.0000 | 0.0000 | 0.0000 |
|  | Alcaligenaceae_unclassified | 8.02E-05 | 0.0002 | 0.0003 | 0.0000 | 8.02E-05 | 8.02E-05 |  | Guehomyces | 0.0000 | 0.0000 | 0.0000 | 0.0002 | 0.0000 | 0.0000 |
|  | Alcanivorax | 0.0006 | 0.0006 | 0.0000 | 0.0000 | 0.0000 | 0.0000 |  | Incertae_sedis_unclassified | 0.0007 | 0.0009 | 0.0005 | 0.0031 | 0.0002 | 0.0007 |
|  | Alistipes | 0.0032 | 0.0030 | 8.02E-05 | 0.0000 | 0.0000 | 0.0002 |  | Incertae_sedis_unidentified | 0.0000 | 0.0000 | 0.0000 | 0.0014 | 0.0000 | 0.0000 |
|  | Alkaliphilus | 0.0004 | 0.0006 | 0.0053 | 0.0002 | 0.0054 | 0.0064 |  | Lactarius | 0.0000 | 0.0000 | 0.0000 | 0.0003 | 0.0000 | 0.0000 |
|  | Alphaproteobacteria_unclassified | 0.0010 | 0.0018 | 0.0000 | 0.0000 | 0.0000 | 0.0000 |  | Leotiomycetes_unclassified | 0.0000 | 0.0000 | 8.26E-05 | 0.0000 | 0.0000 | 0.0000 |
|  | Anaerolineaceae_unclassified | 0.0005 | 8.02E-05 | 0.0000 | 0.0000 | 0.0000 | 0.0000 |  | Lophiostoma | 0.0000 | 0.0000 | 0.0000 | 0.0065 | 0.0000 | 0.0000 |
|  | Anaerolineaceae_uncultured | 0.0061 | 0.0062 | 0.0003 | 8.02E-05 | 0.0000 | 0.0010 |  | Malassezia | 0.0000 | 0.0000 | 0.0000 | 0.0003 | 0.0000 | 0.0000 |
|  | Anaerostipes | 0.0034 | 0.0038 | 0.0002 | 0.0000 | 0.0000 | 0.0002 |  | Mortierella | 0.0000 | 0.0000 | 0.0000 | 0.0003 | 0.0000 | 0.0000 |
|  | Anderseniella | 0.0014 | 0.0009 | 0.0000 | 0.0000 | 0.0000 | 0.0000 |  | Mycosphaerella | 0.0000 | 0.0265 | 0.0000 | 0.0000 | 0.0000 | 0.0002 |
|  | Aquibacter | 0.0002 | 0.0002 | 0.0000 | 0.0000 | 0.0000 | 0.0000 |  | Mycosphaerellaceae_unclassified | 0.0000 | 0.0000 | 0.0004 | 0.0000 | 4.13E-05 | 0.0000 |
|  | Ardenticatenia_uncultured | 0.0060 | 0.0055 | 0.0000 | 0.0000 | 0.0000 | 0.0000 |  | Ophiocordycipitaceae_unclassified | 0.0572 | 0.3356 | 0.3366 | 0.1786 | 0.0699 | 0.0931 |
|  | Arsenophonus | 0.0000 | 0.0000 | 0.0000 | 0.9941 | 0.9099 | 0.0037 |  | Penicillium | 0.0005 | 0.0223 | 0.0005 | 0.0019 | 0.0001 | 0.0173 |
|  | B1-7BS_norank | 0.0006 | 0.0004 | 0.0000 | 8.02E-05 | 0.0000 | 0.0000 |  | Peniophora | 0.0000 | 0.0000 | 0.0000 | 8.26E-05 | 0.0000 | 0.0000 |
|  | BD1-7_clade | 0.0000 | 0.0000 | 0.0000 | 0.0000 | 0.0000 | 0.0002 |  | Phaeosphaeriaceae_unclassified | 0.0000 | 4.13E-05 | 0.0003 | 0.0000 | 4.13E-05 | 4.13E-05 |
|  | BD2-11_terrestrial_group_norank | 0.0006 | 0.0005 | 0.0000 | 0.0000 | 0.0000 | 0.0000 |  | Pleosporaceae_unclassified | 0.0001 | 0.0000 | 0.0000 | 0.0000 | 0.0000 | 0.0000 |
|  | BD7-8_marine_group_norank | 0.0177 | 0.0205 | 0.0000 | 0.0000 | 0.0000 | 0.0000 |  | Pleosporales_unclassified | 0.0000 | 0.0000 | 0.0000 | 0.0002 | 0.0000 | 0.0003 |
|  | BSV26_norank | 0.0006 | 0.0006 | 0.0000 | 0.0000 | 0.0000 | 0.0000 |  | Podosphaera | 0.0000 | 0.0000 | 8.26E-05 | 0.0000 | 0.0000 | 0.0000 |
|  | Bacillus | 0.0042 | 0.0028 | 0.0410 | 0.0005 | 0.0322 | 0.0412 |  | Rachicladosporium | 0.0000 | 0.0000 | 0.0002 | 0.0001 | 4.13E-05 | 4.13E-05 |
|  | Bacteria_unclassified | 0.0015 | 0.0017 | 0.0000 | 0.0000 | 0.0000 | 0.0000 |  | Rhodotorula | 0.0000 | 0.0000 | 0.0000 | 0.0003 | 4.13E-05 | 0.0000 |
|  | Bacteroides | 0.0219 | 0.0179 | 0.0003 | 0.0001 | 0.0002 | 0.0016 |  | Russula | 0.0000 | 0.0000 | 0.0000 | 0.0004 | 0.0000 | 0.0000 |
|  | Bacteroidetes_BD2-2_norank | 0.0179 | 0.0184 | 0.0000 | 0.0000 | 0.0000 | 0.0000 |  | Sarcinomyces | 0.0000 | 0.0000 | 0.0000 | 0.0003 | 0.0000 | 0.0000 |
|  | Barnesiella | 0.0002 | 0.0010 | 0.0000 | 0.0000 | 0.0000 | 0.0000 |  | Schizophyllum | 0.0000 | 4.13E-05 | 0.0000 | 8.26E-05 | 0.0000 | 0.0000 |
|  | Bifidobacterium | 0.0071 | 0.0107 | 0.0000 | 0.0000 | 0.0000 | 0.0009 |  | Sclerotiniaceae_unclassified | 0.0000 | 0.0000 | 0.0003 | 0.0002 | 0.0000 | 0.0000 |
|  | Blautia | 0.0105 | 0.0123 | 0.0000 | 0.0000 | 8.02E-05 | 0.0006 |  | Sordariomycetes_unclassified | 0.0172 | 0.0016 | 0.0016 | 0.0043 | 0.0002 | 8.26E-05 |
|  | Bosea | 0.0003 | 8.02E-05 | 0.0006 | 0.0000 | 0.0007 | 8.02E-05 |  | Spegazzinia | 0.0000 | 0.0000 | 0.0000 | 0.0000 | 0.0000 | 8.26E-05 |
|  | Brevibacillus | 0.0000 | 0.0000 | 0.0008 | 0.0000 | 0.0005 | 0.0006 |  | Spencermartinsia | 8.26E-05 | 0.0000 | 0.0000 | 0.0000 | 8.26E-05 | 0.0000 |
|  | CA002_norank | 0.0010 | 0.0008 | 0.0000 | 0.0000 | 0.0000 | 0.0000 |  | Sporobolomyces | 0.0000 | 0.0000 | 0.0000 | 8.26E-05 | 0.0000 | 0.0000 |
|  | Caldilineaceae_uncultured | 0.0043 | 0.0052 | 0.0000 | 0.0000 | 0.0000 | 0.0000 |  | Stachybotrys | 0.0000 | 0.0000 | 0.0000 | 0.0000 | 0.0000 | 8.26E-05 |
|  | Caldithrix | 0.0039 | 0.0038 | 0.0000 | 0.0000 | 0.0000 | 0.0000 |  | Strelitziana | 0.0000 | 0.0000 | 0.0001 | 8.26E-05 | 0.0000 | 4.13E-05 |
|  | Candidatus_Acetothermus | 0.0002 | 8.02E-05 | 0.0000 | 0.0000 | 0.0000 | 0.0000 |  | Sympodiomycopsis | 4.13E-05 | 0.0000 | 4.13E-05 | 0.0003 | 0.0000 | 0.0005 |
|  | Candidatus_Entotheonella | 0.0037 | 0.0050 | 0.0000 | 0.0000 | 0.0000 | 0.0000 |  | Teratosphaeria | 4.13E-05 | 4.13E-05 | 0.0016 | 0.0006 | 0.0158 | 8.26E-05 |
|  | Candidatus_Microthrix | 8.02E-05 | 0.0000 | 0.0015 | 0.0000 | 0.0017 | 0.0010 |  | Teratosphaeriaceae_unclassified | 0.0000 | 8.26E-05 | 0.0002 | 0.0000 | 0.0037 | 4.13E-05 |
|  | Candidatus_Protochlamydia | 0.0000 | 0.0000 | 0.0000 | 0.0000 | 8.02E-05 | 0.0000 |  | Teratosphaeriaceae_unidentified | 0.0000 | 0.0000 | 8.26E-05 | 0.0000 | 0.0000 | 0.0000 |
|  | Carnobacterium | 0.0000 | 0.0000 | 0.0000 | 0.0000 | 0.0005 | 0.0004 |  | Tilletiopsis | 4.13E-05 | 0.0002 | 4.13E-05 | 4.13E-05 | 4.13E-05 | 0.0000 |
|  | Cellvibrionaceae_uncultured | 0.0002 | 8.02E-05 | 0.0000 | 0.0000 | 0.0000 | 0.0000 |  | Toxicocladosporium | 0.0000 | 4.13E-05 | 0.0002 | 0.0000 | 0.0000 | 0.0001 |
|  | Chitinophagaceae_unclassified | 0.0000 | 8.02E-05 | 0.0000 | 0.0000 | 8.02E-05 | 0.0000 |  | Tremellales_unclassified | 0.0000 | 0.0000 | 4.13E-05 | 0.0000 | 4.13E-05 | 0.0002 |
|  | Chitinophagaceae_uncultured | 0.0000 | 8.02E-05 | 0.0003 | 0.0000 | 0.0002 | 0.0010 |  | Trichothecium | 0.0000 | 0.0000 | 0.0000 | 0.0214 | 0.0000 | 0.0000 |
|  | Chloroflexi_uncultured | 0.0008 | 0.0006 | 0.0000 | 0.0000 | 0.0000 | 0.0000 |  | Umbelopsis | 0.0000 | 0.0000 | 0.0000 | 0.0002 | 0.0000 | 0.0000 |
|  | Christensenellaceae_R-7_group | 0.0006 | 0.0003 | 0.0000 | 0.0000 | 0.0000 | 0.0000 |  | Valsaceae_unidentified | 0.0000 | 0.0000 | 0.0000 | 8.26E-05 | 0.0000 | 0.0000 |
|  | Chromatiales_unclassified | 8.02E-05 | 0.0003 | 0.0000 | 0.0000 | 0.0000 | 0.0000 |  | Wallrothiella | 0.0000 | 0.0002 | 8.26E-05 | 0.0000 | 4.13E-05 | 0.0001 |
|  | Clostridium_sensu_stricto_18 | 0.0002 | 0.0000 | 0.0003 | 0.0000 | 0.0002 | 0.0006 |  |  |  |  |  |  |  |  |
|  | Collinsella | 0.0002 | 0.0000 | 0.0000 | 0.0000 | 0.0002 | 8.02E-05 |  |  |  |  |  |  |  |  |
|  | Cronobacter | 0.0004 | 0.0002 | 0.0050 | 0.0000 | 0.0051 | 0.0046 |  |  |  |  |  |  |  |  |
|  | Cyanobacteria_norank | 0.0036 | 0.0041 | 0.0000 | 0.0006 | 0.0030 | 0.0035 |  |  |  |  |  |  |  |  |
|  | Cytophagaceae_unclassified | 0.0000 | 0.0000 | 0.0000 | 0.0000 | 0.0000 | 0.0003 |  |  |  |  |  |  |  |  |
|  | Cytophagaceae_uncultured | 0.0000 | 0.0000 | 0.0000 | 0.0000 | 0.0002 | 0.0006 |  |  |  |  |  |  |  |  |
|  | Deferrisoma | 0.0029 | 0.0023 | 0.0000 | 0.0000 | 0.0000 | 0.0000 |  |  |  |  |  |  |  |  |
|  | Defluviicoccus | 0.0016 | 0.0022 | 0.0000 | 0.0000 | 0.0000 | 0.0000 |  |  |  |  |  |  |  |  |
|  | Dehalobium | 0.0007 | 0.0006 | 0.0000 | 0.0000 | 0.0000 | 0.0000 |  |  |  |  |  |  |  |  |
|  | Dehalococcoidia_unclassified | 0.0004 | 0.0002 | 0.0000 | 0.0000 | 0.0000 | 0.0000 |  |  |  |  |  |  |  |  |
|  | Deltaproteobacteria_unclassified | 0.0035 | 0.0023 | 0.0000 | 0.0000 | 0.0000 | 0.0000 |  |  |  |  |  |  |  |  |
|  | Desulfatiglans | 0.0042 | 0.0035 | 0.0000 | 0.0000 | 0.0000 | 0.0000 |  |  |  |  |  |  |  |  |
|  | Desulfobacteraceae_unclassified | 0.0005 | 0.0009 | 0.0000 | 0.0000 | 0.0000 | 0.0000 |  |  |  |  |  |  |  |  |
|  | Desulfobulbaceae_unclassified | 0.0014 | 0.0018 | 0.0000 | 0.0000 | 0.0000 | 0.0000 |  |  |  |  |  |  |  |  |
|  | Desulfobulbaceae_uncultured | 0.0057 | 0.0046 | 0.0000 | 0.0000 | 0.0000 | 0.0000 |  |  |  |  |  |  |  |  |
|  | Desulfobulbus | 0.0364 | 0.0293 | 0.0000 | 0.0000 | 0.0000 | 0.0000 |  |  |  |  |  |  |  |  |
|  | Desulfococcus | 0.0022 | 0.0019 | 0.0000 | 0.0000 | 0.0000 | 0.0003 |  |  |  |  |  |  |  |  |
|  | Desulfosarcina | 0.0010 | 0.0012 | 0.0000 | 0.0000 | 0.0000 | 0.0000 |  |  |  |  |  |  |  |  |
|  | Desulfurivibrio | 0.0028 | 0.0026 | 0.0000 | 0.0000 | 0.0000 | 0.0000 |  |  |  |  |  |  |  |  |
|  | Dialister | 8.02E-05 | 0.0004 | 0.0000 | 0.0000 | 0.0000 | 0.0000 |  |  |  |  |  |  |  |  |
|  | Dorea | 0.0008 | 0.0010 | 0.0000 | 0.0000 | 0.0000 | 0.0000 |  |  |  |  |  |  |  |  |
|  | Draconibacterium | 0.0011 | 0.0012 | 0.0000 | 0.0000 | 0.0000 | 0.0000 |  |  |  |  |  |  |  |  |
|  | Ectothiorhodospiraceae_norank | 0.0042 | 0.0040 | 0.0000 | 0.0000 | 0.0000 | 0.0000 |  |  |  |  |  |  |  |  |
|  | Enterococcus | 0.0002 | 0.0003 | 0.0022 | 0.0000 | 0.0023 | 0.0034 |  |  |  |  |  |  |  |  |
|  | Erysipelotrichaceae_UCG-003 | 0.0005 | 0.0003 | 0.0000 | 0.0000 | 0.0000 | 8.02E-05 |  |  |  |  |  |  |  |  |
|  | Escherichia-Shigella | 0.0002 | 0.0002 | 0.0014 | 0.0005 | 0.0014 | 0.0029 |  |  |  |  |  |  |  |  |
|  | Eudoraea | 0.0047 | 0.0040 | 0.0000 | 0.0000 | 0.0000 | 0.0000 |  |  |  |  |  |  |  |  |
|  | Exiguobacterium | 8.02E-05 | 0.0000 | 8.02E-05 | 0.0000 | 0.0002 | 0.0000 |  |  |  |  |  |  |  |  |
|  | FFCH9454_norank | 0.0002 | 0.0002 | 0.0000 | 0.0000 | 0.0000 | 0.0000 |  |  |  |  |  |  |  |  |
|  | FW22_norank | 0.0002 | 0.0002 | 0.0000 | 0.0000 | 0.0000 | 0.0000 |  |  |  |  |  |  |  |  |
|  | Faecalibacterium | 0.0182 | 0.0209 | 8.02E-05 | 0.0000 | 0.0004 | 0.0017 |  |  |  |  |  |  |  |  |
|  | Ferribacterium | 0.0002 | 0.0004 | 0.0004 | 0.0000 | 0.0004 | 0.0015 |  |  |  |  |  |  |  |  |
|  | Fibrobacteraceae_uncultured | 8.02E-05 | 0.0002 | 0.0000 | 0.0000 | 0.0000 | 0.0000 |  |  |  |  |  |  |  |  |
|  | Filomicrobium | 0.0027 | 0.0020 | 0.0000 | 0.0000 | 0.0000 | 0.0000 |  |  |  |  |  |  |  |  |
|  | Flammeovirgaceae_uncultured | 0.0095 | 0.0092 | 0.0000 | 0.0000 | 0.0000 | 0.0000 |  |  |  |  |  |  |  |  |
|  | Flavobacteriaceae_unclassified | 0.0011 | 0.0008 | 0.0000 | 0.0000 | 0.0000 | 0.0000 |  |  |  |  |  |  |  |  |
|  | Flavobacteriaceae_uncultured | 0.0003 | 0.0003 | 0.0000 | 0.0000 | 0.0000 | 0.0000 |  |  |  |  |  |  |  |  |
|  | Flavobacterium | 0.0000 | 8.02E-05 | 0.0003 | 0.0000 | 0.0000 | 0.0002 |  |  |  |  |  |  |  |  |
|  | Fluviicola | 0.0003 | 0.0002 | 0.0000 | 0.0000 | 0.0000 | 0.0000 |  |  |  |  |  |  |  |  |
|  | Formosa | 0.0003 | 0.0003 | 0.0000 | 0.0000 | 0.0000 | 0.0000 |  |  |  |  |  |  |  |  |
|  | FukuN18_freshwater_group_norank | 8.02E-05 | 0.0003 | 0.0025 | 0.0000 | 0.0026 | 0.0028 |  |  |  |  |  |  |  |  |
|  | Fusicatenibacter | 0.0000 | 0.0000 | 8.02E-05 | 0.0000 | 0.0000 | 0.0000 |  |  |  |  |  |  |  |  |
|  | Fusobacterium | 0.0000 | 0.0000 | 0.0000 | 0.0000 | 0.0000 | 0.0004 |  |  |  |  |  |  |  |  |
|  | GKS98_freshwater_group | 0.0010 | 0.0009 | 0.0000 | 0.0000 | 0.0000 | 0.0000 |  |  |  |  |  |  |  |  |
|  | GR-WP33-30_norank | 0.0002 | 0.0006 | 0.0000 | 0.0000 | 0.0000 | 0.0000 |  |  |  |  |  |  |  |  |
|  | Gaiellales_uncultured | 0.0014 | 0.0014 | 0.0000 | 0.0000 | 0.0000 | 0.0000 |  |  |  |  |  |  |  |  |
|  | Gammaproteobacteria_unclassified | 0.0067 | 0.0067 | 0.0000 | 0.0000 | 0.0000 | 0.0000 |  |  |  |  |  |  |  |  |
|  | Gemmatimonadaceae_norank | 0.0007 | 0.0015 | 0.0000 | 0.0000 | 0.0002 | 0.0000 |  |  |  |  |  |  |  |  |
|  | Gemmatimonadaceae_uncultured | 0.0032 | 0.0026 | 0.0000 | 0.0000 | 0.0000 | 8.02E-05 |  |  |  |  |  |  |  |  |
|  | Geobacter | 8.02E-05 | 0.0000 | 0.0000 | 0.0000 | 0.0000 | 0.0000 |  |  |  |  |  |  |  |  |
|  | Geobacteraceae_norank | 0.0003 | 0.0002 | 0.0000 | 0.0000 | 0.0000 | 0.0000 |  |  |  |  |  |  |  |  |
|  | Granulosicoccus | 0.0007 | 0.0011 | 0.0000 | 0.0000 | 0.0000 | 0.0000 |  |  |  |  |  |  |  |  |
|  | HOC36_norank | 0.0096 | 0.0103 | 0.0000 | 0.0000 | 0.0000 | 0.0000 |  |  |  |  |  |  |  |  |
|  | Haliangium | 0.0000 | 0.0000 | 0.0000 | 0.0000 | 0.0000 | 0.0002 |  |  |  |  |  |  |  |  |
|  | Haliea | 0.0044 | 0.0041 | 0.0000 | 0.0000 | 0.0000 | 0.0000 |  |  |  |  |  |  |  |  |
|  | Halieaceae_unclassified | 0.0032 | 0.0029 | 0.0000 | 0.0000 | 0.0000 | 0.0000 |  |  |  |  |  |  |  |  |
|  | Halioglobus | 0.0010 | 0.0015 | 0.0000 | 0.0000 | 0.0000 | 0.0000 |  |  |  |  |  |  |  |  |
|  | Haliscomenobacter | 0.0000 | 0.0000 | 8.02E-05 | 0.0000 | 8.02E-05 | 0.0002 |  |  |  |  |  |  |  |  |
|  | Halomonas | 0.0002 | 0.0002 | 0.0000 | 0.0000 | 0.0000 | 0.0000 |  |  |  |  |  |  |  |  |
|  | Hydrogenophilaceae_uncultured | 0.0011 | 0.0008 | 8.02E-05 | 0.0000 | 0.0000 | 0.0000 |  |  |  |  |  |  |  |  |
|  | Hyphomicrobiaceae_uncultured | 0.0092 | 0.0110 | 0.0000 | 8.02E-05 | 0.0000 | 0.0000 |  |  |  |  |  |  |  |  |
|  | Illumatobacter | 0.0036 | 0.0029 | 0.0000 | 0.0000 | 0.0000 | 0.0000 |  |  |  |  |  |  |  |  |
|  | Intestinibacter | 0.0002 | 8.02E-05 | 0.0000 | 0.0000 | 0.0000 | 0.000160308 |  |  |  |  |  |  |  |  |
|  | Intrasporangiaceae_unclassified | 0.0002 | 0.0000 | 0.001042001 | 0.000160308 | 0.000561077 | 0.000881693 |  |  |  |  |  |  |  |  |
|  | JL-ETNP-Z39_norank | 0.0005 | 0.0006 | 0.0000 | 0.0000 | 0.0000 | 0.0000 |  |  |  |  |  |  |  |  |
|  | JTB255_marine_benthic_group_norank | 0.0545 | 0.0522 | 0.0000 | 8.02E-05 | 0.0000 | 0.0000 |  |  |  |  |  |  |  |  |
|  | KD4-96_norank | 0.0079 | 0.0062 | 0.0000 | 0.0000 | 0.0000 | 0.0000 |  |  |  |  |  |  |  |  |
|  | KI89A_clade_norank | 0.0044 | 0.0061 | 0.0000 | 0.0000 | 0.0000 | 0.0000 |  |  |  |  |  |  |  |  |
|  | Kluyvera | 0.0026 | 0.0029 | 8.02E-05 | 8.02E-05 | 0.0000 | 0.0004 |  |  |  |  |  |  |  |  |
|  | Kocuria | 0.0000 | 0.0000 | 8.02E-05 | 0.0000 | 0.0002 | 8.02E-05 |  |  |  |  |  |  |  |  |
|  | Lachnoclostridium | 0.0009 | 0.0006 | 0.0002 | 0.0000 | 0.0000 | 0.0000 |  |  |  |  |  |  |  |  |
|  | Lachnospiraceae_NK4A136_group | 0.0005 | 0.0006 | 0.0000 | 0.0000 | 0.0000 | 0.0000 |  |  |  |  |  |  |  |  |
|  | Lachnospiraceae_unclassified | 0.0014 | 0.0021 | 0.0000 | 0.0000 | 0.0000 | 0.0000 |  |  |  |  |  |  |  |  |
|  | Lachnospiraceae_uncultured | 0.0003 | 0.0002 | 0.0000 | 0.0000 | 0.0000 | 0.0000 |  |  |  |  |  |  |  |  |
|  | Lactococcus | 8.02E-05 | 8.02E-05 | 0.0010 | 0.0000 | 0.0014 | 0.0017 |  |  |  |  |  |  |  |  |
|  | Latescibacteria_norank | 0.0031 | 0.0019 | 0.0000 | 0.0000 | 0.0000 | 0.0000 |  |  |  |  |  |  |  |  |
|  | Leptospiraceae_uncultured | 0.0006 | 0.0005 | 0.0000 | 0.0000 | 0.0000 | 0.0000 |  |  |  |  |  |  |  |  |
|  | Lutimonas | 0.0055 | 0.0045 | 0.0000 | 0.0000 | 0.0000 | 0.0000 |  |  |  |  |  |  |  |  |
|  | MBAE14_norank | 0.0003 | 0.0003 | 0.0000 | 0.0000 | 0.0000 | 0.0000 |  |  |  |  |  |  |  |  |
|  | MSB-1E8_norank | 0.0111 | 0.0087 | 0.0000 | 0.0000 | 0.0000 | 0.0000 |  |  |  |  |  |  |  |  |
|  | MSBL5_norank | 0.0002 | 0.0002 | 0.0000 | 0.0000 | 0.0000 | 0.0000 |  |  |  |  |  |  |  |  |
|  | MSBL7 | 0.0008 | 0.0010 | 0.0000 | 0.0000 | 0.0000 | 0.0000 |  |  |  |  |  |  |  |  |
|  | Marinicella | 0.0195 | 0.0184 | 0.0000 | 0.0000 | 0.0000 | 0.0000 |  |  |  |  |  |  |  |  |
|  | Marinobacterium | 0.0002 | 8.02E-05 | 0.0000 | 0.0000 | 0.0000 | 0.0000 |  |  |  |  |  |  |  |  |
|  | Mariprofundus | 0.0002 | 8.02E-05 | 0.0000 | 0.0000 | 0.0000 | 0.0000 |  |  |  |  |  |  |  |  |
|  | MidBa8_norank | 0.0008 | 0.0010 | 0.0000 | 0.0000 | 0.0000 | 0.0000 |  |  |  |  |  |  |  |  |
|  | Moraxella | 0.0006 | 0.0002 | 0.0000 | 0.0000 | 8.02E-05 | 0.0000 |  |  |  |  |  |  |  |  |
|  | NKB5_norank | 0.0006 | 0.0002 | 0.0000 | 0.0000 | 0.0000 | 0.0000 |  |  |  |  |  |  |  |  |
|  | NS72_norank | 0.0006 | 0.0002 | 0.0000 | 0.0000 | 0.0000 | 0.0000 |  |  |  |  |  |  |  |  |
|  | Neptunomonas | 0.0009 | 0.0006 | 0.0000 | 0.0000 | 0.0000 | 0.0000 |  |  |  |  |  |  |  |  |
|  | Nesterenkonia | 0.0000 | 0.0000 | 8.02E-05 | 0.0000 | 0.0002 | 0.0000 |  |  |  |  |  |  |  |  |
|  | Nitrosococcus | 0.0313 | 0.0302 | 0.0000 | 0.0000 | 0.0000 | 0.0000 |  |  |  |  |  |  |  |  |
|  | Nitrosomonas | 0.0018 | 0.0011 | 0.0000 | 0.0000 | 0.0000 | 0.0000 |  |  |  |  |  |  |  |  |
|  | Nitrospinaceae_uncultured | 0.0046 | 0.0038 | 0.0000 | 0.0000 | 0.0000 | 0.0000 |  |  |  |  |  |  |  |  |
|  | Nitrospira | 0.0701 | 0.0701 | 0.0006 | 0.0000 | 0.0003 | 0.0002 |  |  |  |  |  |  |  |  |
|  | OCS116_clade_norank | 0.0002 | 0.0002 | 0.0000 | 0.0000 | 0.0000 | 0.0000 |  |  |  |  |  |  |  |  |
|  | OM182_clade_norank | 0.0003 | 0.0004 | 0.0000 | 0.0000 | 0.0000 | 0.0000 |  |  |  |  |  |  |  |  |
|  | OM190_norank | 0.0008 | 0.0018 | 0.0000 | 0.0000 | 0.0000 | 0.0000 |  |  |  |  |  |  |  |  |
|  | OM1_clade_norank | 0.0372 | 0.0402 | 0.0000 | 0.0000 | 0.0000 | 0.0000 |  |  |  |  |  |  |  |  |
|  | OM60_NOR5_clade | 0.0004 | 0.0004 | 0.0000 | 0.0000 | 0.0000 | 0.0000 |  |  |  |  |  |  |  |  |
|  | OPB35_soil_group_norank | 0.0000 | 0.0000 | 8.02E-05 | 0.0000 | 0.0000 | 0.0000 |  |  |  |  |  |  |  |  |
|  | Obscuribacterales_norank | 0.0000 | 0.0000 | 0.0003 | 8.02E-05 | 8.02E-05 | 0.0000 |  |  |  |  |  |  |  |  |
|  | Oceanobacillus | 0.0000 | 8.02E-05 | 0.0002 | 0.0000 | 0.0004 | 0.0005 |  |  |  |  |  |  |  |  |
|  | Oligoflexaceae_norank | 8.02E-05 | 8.02E-05 | 0.0000 | 0.0000 | 0.0000 | 0.0000 |  |  |  |  |  |  |  |  |
|  | Oligoflexales_norank | 0.0002 | 0.0003 | 0.0000 | 0.0000 | 0.0000 | 0.0000 |  |  |  |  |  |  |  |  |
|  | Opitutae_unclassified | 0.0008 | 0.0008 | 0.0000 | 0.0000 | 0.0000 | 0.0000 |  |  |  |  |  |  |  |  |
|  | Ottowia | 0.0012 | 0.0003 | 0.0000 | 8.02E-05 | 0.0003 | 0.0002 |  |  |  |  |  |  |  |  |
|  | PAUC43f_marine_benthic_group_norank | 0.0147 | 0.0143 | 0.0000 | 0.0000 | 0.0000 | 0.0000 |  |  |  |  |  |  |  |  |
|  | PHOS-HE36_norank | 0.0059 | 0.0060 | 0.0000 | 8.02E-05 | 8.02E-05 | 8.02E-05 |  |  |  |  |  |  |  |  |
|  | PHOS-HE51_norank | 0.0000 | 0.0000 | 0.0000 | 0.0000 | 0.0000 | 8.02E-05 |  |  |  |  |  |  |  |  |
|  | PS-B29_norank | 0.0006 | 0.0006 | 0.0000 | 0.0000 | 0.0000 | 0.0000 |  |  |  |  |  |  |  |  |
|  | Paenibacillus | 0.0012 | 0.0018 | 0.0258 | 8.02E-05 | 0.0223 | 0.0227 |  |  |  |  |  |  |  |  |
|  | Parabacteroides | 0.0006 | 0.0010 | 0.0000 | 0.0000 | 0.0000 | 0.0000 |  |  |  |  |  |  |  |  |
|  | Parasutterella | 0.0008 | 0.0005 | 0.0000 | 0.0000 | 0.0000 | 0.0002 |  |  |  |  |  |  |  |  |
|  | Parcubacteria_norank | 0.0003 | 0.0002 | 0.0000 | 0.0000 | 0.0000 | 0.0003 |  |  |  |  |  |  |  |  |
|  | Pelagibius | 0.0046 | 0.0048 | 0.0000 | 0.0000 | 0.0000 | 0.0000 |  |  |  |  |  |  |  |  |
|  | Phascolarctobacterium | 0.0002 | 0.0004 | 0.0000 | 0.0000 | 0.0000 | 0.0000 |  |  |  |  |  |  |  |  |
|  | Phycisphaeraceae_uncultured | 0.0004 | 0.0002 | 0.0000 | 0.0000 | 0.0000 | 0.0000 |  |  |  |  |  |  |  |  |
|  | Phycisphaerales_unclassified | 8.02E-05 | 0.0002 | 0.0000 | 0.0000 | 0.0000 | 0.0000 |  |  |  |  |  |  |  |  |
|  | Pla4_lineage_norank | 0.0008 | 0.0009 | 0.0000 | 0.0000 | 0.0000 | 0.0000 |  |  |  |  |  |  |  |  |
|  | Planctomycetes_unclassified | 0.0002 | 0.0000 | 0.0000 | 0.0000 | 0.0000 | 0.0000 |  |  |  |  |  |  |  |  |
|  | Prevotella_9 | 0.0011 | 0.0009 | 8.02E-05 | 0.0000 | 0.0003 | 0.0002 |  |  |  |  |  |  |  |  |
|  | Prevotellaceae_NK3B31_group | 0.0010 | 0.0010 | 0.0000 | 0.0000 | 0.0000 | 0.0000 |  |  |  |  |  |  |  |  |
|  | Prevotellaceae_UCG-001 | 0.0006 | 0.0003 | 0.0000 | 0.0000 | 0.0000 | 8.02E-05 |  |  |  |  |  |  |  |  |
|  | Prolixibacter | 0.0014 | 0.0010 | 0.0000 | 0.0000 | 0.0000 | 0.0000 |  |  |  |  |  |  |  |  |
|  | Propionigenium | 0.0021 | 0.0015 | 0.0000 | 0.0000 | 0.0000 | 0.0000 |  |  |  |  |  |  |  |  |
|  | Proteobacteria_unclassified | 0.0006 | 0.0007 | 0.0000 | 0.0000 | 0.0000 | 0.0000 |  |  |  |  |  |  |  |  |
|  | Pseudahrensia | 0.0011 | 0.0011 | 0.0000 | 0.0000 | 0.0000 | 0.0000 |  |  |  |  |  |  |  |  |
|  | Pseudoalteromonas | 0.0000 | 0.0002 | 0.0000 | 0.0000 | 0.0000 | 0.0000 |  |  |  |  |  |  |  |  |
|  | Pseudobutyrivibrio | 0.0050 | 0.0059 | 8.02E-05 | 0.0000 | 0.0000 | 0.0006 |  |  |  |  |  |  |  |  |
|  | Pseudomonas | 8.02E-05 | 8.02E-05 | 0.0010 | 0.0018 | 0.0018 | 0.0009 |  |  |  |  |  |  |  |  |
|  | Psychrilyobacter | 0.0010 | 0.0010 | 0.0000 | 0.0000 | 0.0000 | 0.0000 |  |  |  |  |  |  |  |  |
|  | Psychromonas | 0.0009 | 0.0014 | 0.0000 | 0.0000 | 0.0000 | 0.0000 |  |  |  |  |  |  |  |  |
|  | R76-B128_norank | 0.0002 | 8.02E-05 | 0.0000 | 0.0000 | 0.0000 | 0.0000 |  |  |  |  |  |  |  |  |
|  | Ralstonia | 0.0036 | 0.0026 | 0.0000 | 0.0000 | 0.0000 | 0.0000 |  |  |  |  |  |  |  |  |
|  | Raoultella | 0.0002 | 0.0004 | 8.02E-05 | 0.0003 | 0.0002 | 0.0005 |  |  |  |  |  |  |  |  |
|  | Reichenbachiella | 0.0018 | 0.0026 | 0.0000 | 0.0000 | 0.0000 | 0.0000 |  |  |  |  |  |  |  |  |
|  | Rhodobacteraceae_unclassified | 0.0034 | 0.0036 | 0.0000 | 0.0000 | 0.0000 | 0.0000 |  |  |  |  |  |  |  |  |
|  | Rhodococcus | 0.0000 | 0.0000 | 0.0000 | 0.0000 | 0.0000 | 0.0004 |  |  |  |  |  |  |  |  |
|  | Rhodospirillaceae_unclassified | 0.0003 | 0.0004 | 0.0000 | 0.0000 | 0.0000 | 0.0000 |  |  |  |  |  |  |  |  |
|  | Rhodospirillaceae_uncultured | 0.0571 | 0.0562 | 0.0000 | 0.0000 | 0.0000 | 0.0000 |  |  |  |  |  |  |  |  |
|  | Rickettsia | 0.0000 | 0.0000 | 0.9022 | 0.0000 | 0.0000 | 0.8806 |  |  |  |  |  |  |  |  |
|  | Romboutsia | 0.0006 | 0.0005 | 0.0002 | 0.0000 | 0.0002 | 0.0002 |  |  |  |  |  |  |  |  |
|  | Roseburia | 0.0004 | 0.0007 | 8.02E-05 | 0.0000 | 8.02E-05 | 8.02E-05 |  |  |  |  |  |  |  |  |
|  | Ruminiclostridium | 0.0003 | 8.02E-05 | 0.0000 | 0.0000 | 0.0000 | 0.0005 |  |  |  |  |  |  |  |  |
|  | Ruminiclostridium_5 | 8.02E-05 | 8.02E-05 | 0.0000 | 0.0000 | 0.0000 | 0.0000 |  |  |  |  |  |  |  |  |
|  | Ruminiclostridium_6 | 0.0003 | 0.0004 | 0.0000 | 0.0000 | 0.0000 | 0.0000 |  |  |  |  |  |  |  |  |
|  | Ruminococcaceae_UCG-002 | 0.0006 | 0.0005 | 0.0000 | 0.0000 | 0.0000 | 0.0000 |  |  |  |  |  |  |  |  |
|  | Ruminococcaceae_UCG-004 | 0.0004 | 0.0006 | 0.0000 | 0.0000 | 0.0000 | 0.0000 |  |  |  |  |  |  |  |  |
|  | Ruminococcaceae_UCG-013 | 0.0006 | 0.0002 | 0.0000 | 0.0000 | 0.0000 | 0.0000 |  |  |  |  |  |  |  |  |
|  | Ruminococcaceae_UCG-014 | 0.0012 | 0.0017 | 0.0000 | 0.0000 | 0.0000 | 0.0000 |  |  |  |  |  |  |  |  |
|  | Ruminococcus_1 | 0.0010 | 0.0010 | 0.0000 | 0.0000 | 0.0000 | 8.02E-05 |  |  |  |  |  |  |  |  |
|  | Ruminococcus_2 | 0.0014 | 0.0009 | 0.0000 | 0.0000 | 0.0000 | 0.0002 |  |  |  |  |  |  |  |  |
|  | SB-5_norank | 0.0010 | 0.0006 | 0.0000 | 0.0000 | 0.0000 | 0.0000 |  |  |  |  |  |  |  |  |
|  | SC3-20_norank | 0.0002 | 0.0002 | 0.0000 | 0.0000 | 0.0000 | 0.0000 |  |  |  |  |  |  |  |  |
|  | SEEP-SRB1 | 0.0025 | 0.0027 | 0.0000 | 0.0000 | 0.0000 | 0.0000 |  |  |  |  |  |  |  |  |
|  | SEEP-SRB4 | 0.0022 | 0.0008 | 0.0000 | 0.0000 | 0.0000 | 0.0000 |  |  |  |  |  |  |  |  |
|  | SHA-109_norank | 0.0002 | 0.0002 | 0.0000 | 0.0000 | 0.0000 | 0.0000 |  |  |  |  |  |  |  |  |
|  | SJA-149_norank | 0.0000 | 0.0000 | 0.0000 | 0.0000 | 0.0000 | 8.02E-05 |  |  |  |  |  |  |  |  |
|  | SM1A02 | 0.0006 | 0.0002 | 0.0000 | 0.0000 | 0.0000 | 0.0000 |  |  |  |  |  |  |  |  |
|  | SPOTSOCT00m83_norank | 0.0000 | 0.0002 | 0.0000 | 0.0000 | 0.0000 | 0.0000 |  |  |  |  |  |  |  |  |
|  | SS1-B-09-64_norank | 0.0007 | 0.0014 | 0.0000 | 0.0000 | 0.0000 | 0.0000 |  |  |  |  |  |  |  |  |
|  | Sandaracinaceae_uncultured | 0.0410 | 0.0459 | 0.0000 | 0.0000 | 0.0000 | 0.0005 |  |  |  |  |  |  |  |  |
|  | Saprospiraceae_uncultured | 0.0008 | 0.0006 | 0.0013 | 0.0006 | 0.0011 | 0.0011 |  |  |  |  |  |  |  |  |
|  | Sh765B-TzT-29_norank | 0.0802 | 0.0763 | 0.0000 | 0.0000 | 0.0000 | 0.0000 |  |  |  |  |  |  |  |  |
|  | Sphaerochaeta | 0.0000 | 0.0000 | 0.0000 | 0.0000 | 0.0000 | 0.0002 |  |  |  |  |  |  |  |  |
|  | Sphingobium | 0.0000 | 0.0000 | 8.02E-05 | 0.0000 | 0.0000 | 0.0000 |  |  |  |  |  |  |  |  |
|  | Sphingomonas | 0.0000 | 0.0000 | 8.02E-05 | 8.02E-05 | 0.0000 | 8.02E-05 |  |  |  |  |  |  |  |  |
|  | Spirochaeta_2 | 0.0086 | 0.0088 | 0.0000 | 0.0000 | 0.0000 | 0.0000 |  |  |  |  |  |  |  |  |
|  | Spongiibacteraceae_uncultured | 0.0004 | 0.0002 | 0.0000 | 0.0000 | 0.0000 | 0.0000 |  |  |  |  |  |  |  |  |
|  | Staphylococcus | 0.0000 | 0.0000 | 0.0000 | 8.02E-05 | 0.0000 | 0.0004 |  |  |  |  |  |  |  |  |
|  | Streptococcus | 0.0000 | 0.0000 | 0.0000 | 0.0000 | 8.02E-05 | 8.02E-05 |  |  |  |  |  |  |  |  |
|  | Subdoligranulum | 0.0099 | 0.0083 | 0.0002 | 0.0000 | 0.0000 | 0.0002 |  |  |  |  |  |  |  |  |
|  | Subgroup_17_norank | 0.0059 | 0.0047 | 0.0000 | 0.0000 | 0.0000 | 0.0000 |  |  |  |  |  |  |  |  |
|  | Subgroup_21_norank | 0.0025 | 0.0026 | 0.0000 | 0.0000 | 0.0000 | 0.0000 |  |  |  |  |  |  |  |  |
|  | Subgroup_23_norank | 0.0084 | 0.0060 | 0.0000 | 0.0000 | 0.0000 | 0.0000 |  |  |  |  |  |  |  |  |
|  | Subgroup_25_norank | 8.02E-05 | 8.02E-05 | 0.0000 | 0.0000 | 0.0000 | 0.0000 |  |  |  |  |  |  |  |  |
|  | Subgroup_3_unclassified | 0.0012 | 0.0016 | 0.0000 | 0.0000 | 0.0000 | 0.0000 |  |  |  |  |  |  |  |  |
|  | Subgroup_6_norank | 0.0002 | 0.0010 | 0.0000 | 0.0000 | 0.0000 | 0.0000 |  |  |  |  |  |  |  |  |
|  | Subgroup_9_norank | 0.0004 | 0.0002 | 0.0000 | 0.0000 | 0.0000 | 0.0000 |  |  |  |  |  |  |  |  |
|  | Sulfuricurvum | 0.0000 | 0.0000 | 0.0000 | 0.0000 | 0.0003 | 0.0002 |  |  |  |  |  |  |  |  |
|  | Sulfuritalea | 0.0002 | 0.0002 | 0.0000 | 0.0000 | 0.0000 | 0.0000 |  |  |  |  |  |  |  |  |
|  | Sulfurovum | 0.0005 | 0.0007 | 0.0000 | 0.0000 | 0.0000 | 0.0000 |  |  |  |  |  |  |  |  |
|  | Sva0081_sediment_group | 0.0450 | 0.0494 | 0.0000 | 0.0000 | 0.0000 | 0.0000 |  |  |  |  |  |  |  |  |
|  | Sva0485_norank | 0.0059 | 0.0060 | 0.0000 | 0.0000 | 0.0000 | 0.0000 |  |  |  |  |  |  |  |  |
|  | Sva0725_norank | 0.0103 | 0.0120 | 0.0000 | 0.0000 | 0.0000 | 0.0000 |  |  |  |  |  |  |  |  |
|  | Sva0996_marine_group_norank | 0.0032 | 0.0026 | 0.0000 | 0.0000 | 0.0000 | 0.0000 |  |  |  |  |  |  |  |  |
|  | Sva1033_norank | 0.0147 | 0.0155 | 0.0000 | 0.0000 | 0.0000 | 0.0000 |  |  |  |  |  |  |  |  |
|  | Synechococcus | 0.0002 | 0.0006 | 0.0000 | 0.0000 | 0.0000 | 0.0000 |  |  |  |  |  |  |  |  |
|  | Syntrophobacteraceae_uncultured | 0.0216 | 0.0249 | 0.0000 | 0.0000 | 0.0000 | 0.0000 |  |  |  |  |  |  |  |  |
|  | TA18_norank | 8.02E-05 | 8.02E-05 | 8.02E-05 | 0.0000 | 0.0002 | 0.0000 |  |  |  |  |  |  |  |  |
|  | TK10_norank | 0.0002 | 0.0004 | 0.0002 | 0.0000 | 0.0002 | 0.0003 |  |  |  |  |  |  |  |  |
|  | TK34_norank | 0.0053 | 0.0068 | 0.0000 | 0.0000 | 0.0000 | 0.0000 |  |  |  |  |  |  |  |  |
|  | TM6_norank | 8.02E-05 | 8.02E-05 | 0.0000 | 0.0000 | 0.0000 | 0.0000 |  |  |  |  |  |  |  |  |
|  | Terrimonas | 0.0000 | 0.0000 | 0.0003 | 0.0000 | 0.0002 | 0.0003 |  |  |  |  |  |  |  |  |
|  | Thioalkalispira | 0.0003 | 0.0004 | 0.0000 | 0.0000 | 0.0000 | 0.0000 |  |  |  |  |  |  |  |  |
|  | Thiogranum | 0.0010 | 0.0011 | 0.0000 | 0.0000 | 0.0000 | 0.0000 |  |  |  |  |  |  |  |  |
|  | Urania-1B-19_marine_sediment_group | 0.0305 | 0.0309 | 0.0000 | 0.0000 | 0.0000 | 0.0000 |  |  |  |  |  |  |  |  |
|  | V2072-189E03_norank | 0.0008 | 0.0014 | 0.0000 | 0.0000 | 0.0000 | 0.0000 |  |  |  |  |  |  |  |  |
|  | Veillonella | 8.02E-05 | 0.0000 | 0.0003 | 0.0000 | 0.0003 | 0.0006 |  |  |  |  |  |  |  |  |
|  | WCHB1-69_norank | 0.0002 | 8.02E-05 | 0.0006 | 0.0002 | 0.0006 | 0.0005 |  |  |  |  |  |  |  |  |
|  | Xanthomonadales_uncultured | 0.0005 | 0.0015 | 0.0000 | 0.0000 | 0.0000 | 8.02E-05 |  |  |  |  |  |  |  |  |
|  | [Eubacterium]_hallii_group | 0.0007 | 0.0016 | 0.0000 | 0.0000 | 0.0000 | 0.0003 |  |  |  |  |  |  |  |  |
|  | possible_order_07_norank | 0.0003 | 0.0000 | 0.0000 | 0.0000 | 0.0000 | 0.0000 |  |  |  |  |  |  |  |  |
|  | vadinHA49_norank | 0.0025 | 0.0015 | 0.0000 | 0.0000 | 0.0000 | 0.0000 |  |  |  |  |  |  |  |  |

**Figure Supplementary materials:**

**
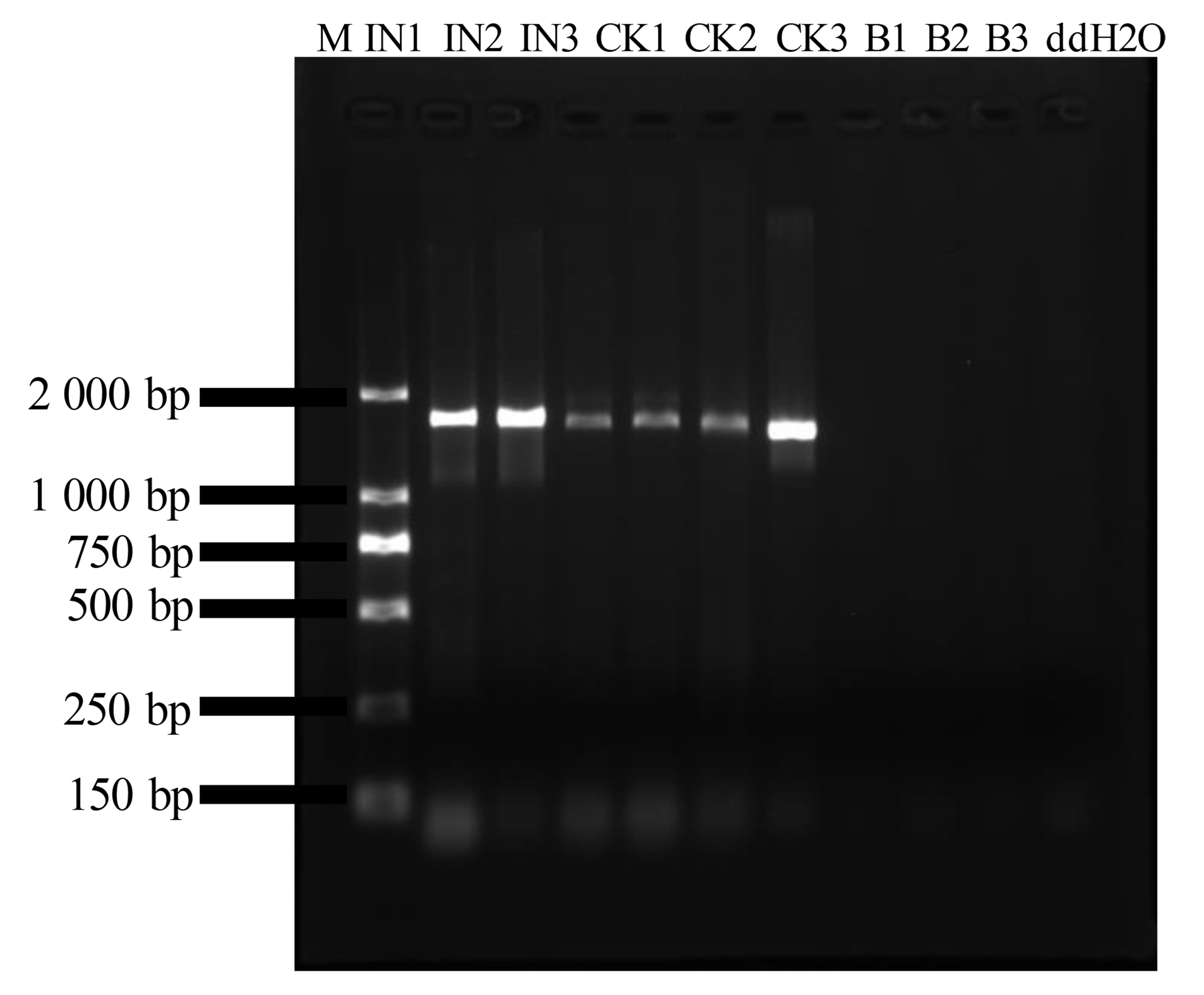
**

**Figure S1. The electrophoresis result of 16S rDNA gene PCR amplification of three bacterial samples.** The lanes from left to right are marker, IN1, IN2, IN3, CK1, CK2, CK3, B1, B2, B3, and ddH_2_O.


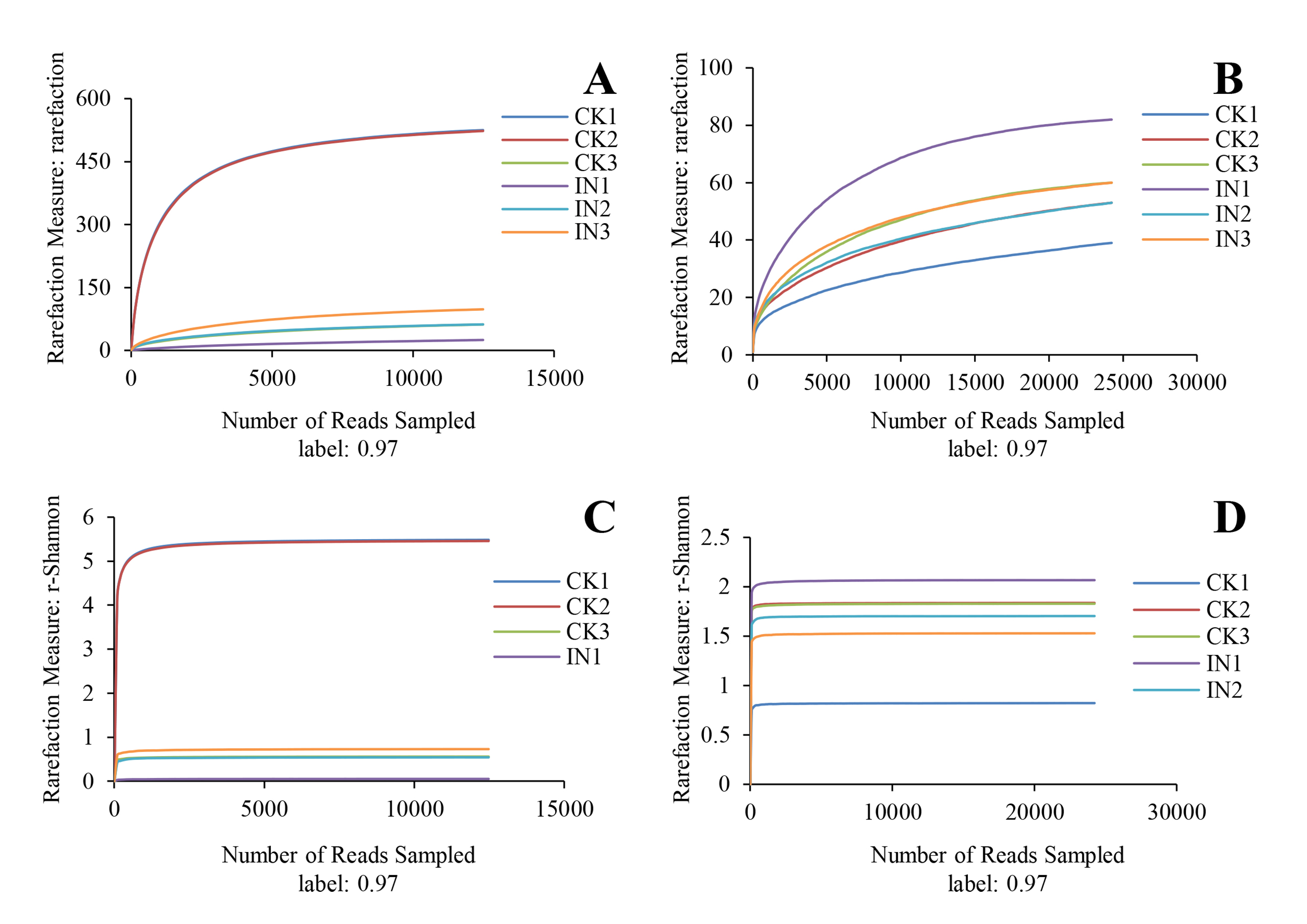


**Figure S2. The rarefaction curve and Shannon-Wiener curve. (A)**, **(B)** The bacterial and fungal rarefaction curve of six samples. **(C)**, **(D)** The bacterial and fungal Shannon-Wiener curve of six samples.


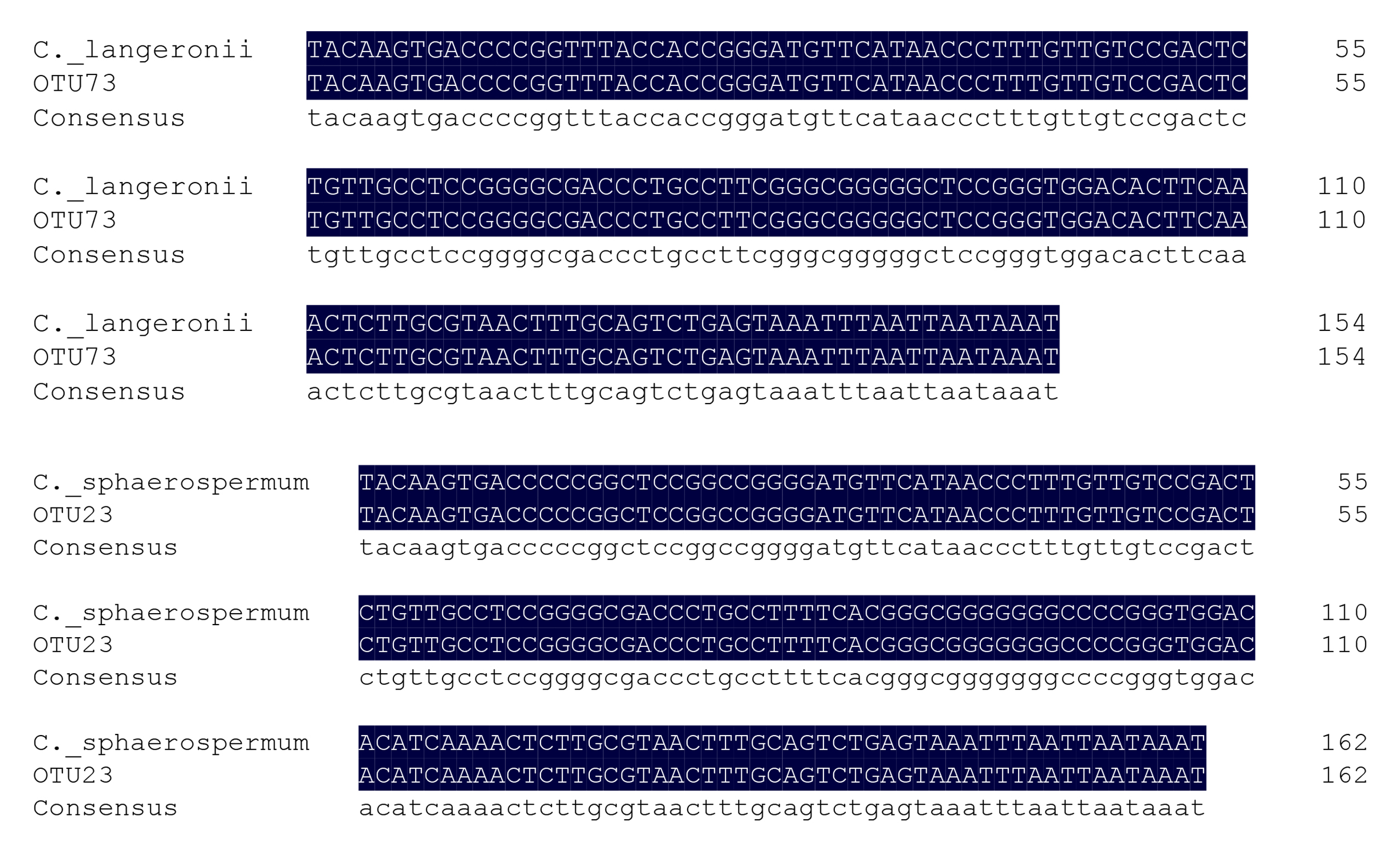


**Figure S3. The ITS I sequences of *C. langeronii* and *C. sphaerospermum* were aligned with OTUs, which were classified into the *Cladosporium* genus.**


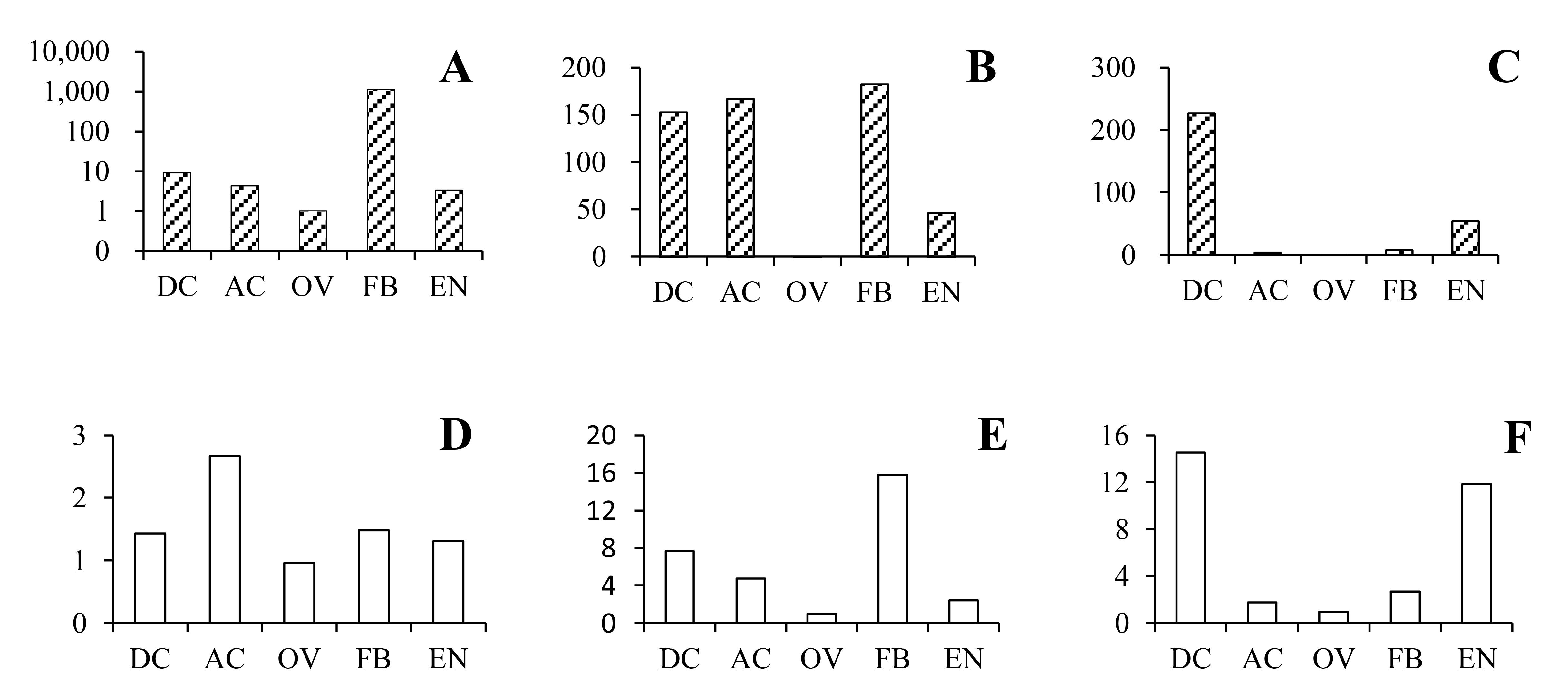


**Figure S4. The relative RT-qPCR results of *C. langeronii* and *C. sphaerospermum* in different tissues of the infected females.** A, B, C: The *C. langeronii* distribution in three infected female samples; D, E, F: The *C. sphaerospermum* distribution in three infected female samples. DC: dorsal cuticle; AC: abdominal cuticle; OV: ovary; FB: fat body; EN: enteron.


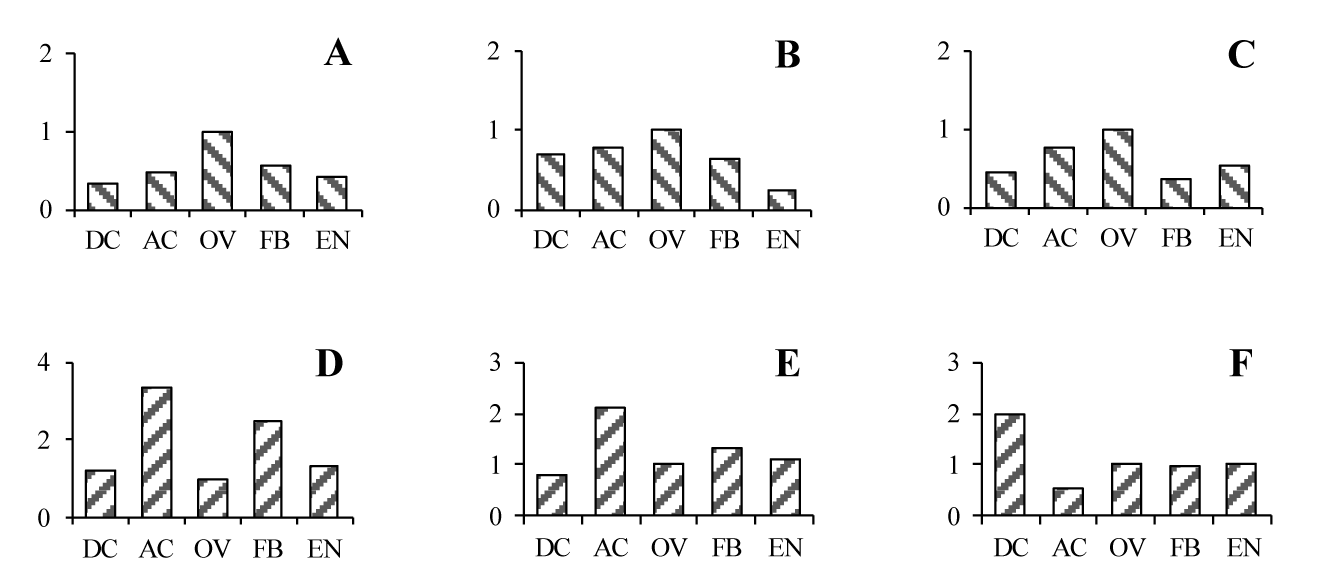


**Figure S5. The relative RT-qPCR results of *C. langeronii* and *C. sphaerospermum* in different tissues of the normal females.** A, B, C: The *C. langeronii* distribution in three normal female samples; D, E, F: The *C. sphaerospermum* distribution in three normal female samples. DC: dorsal cuticle; AC: abdominal cuticle; OV: ovary; FB: fat body; EN: enteron.


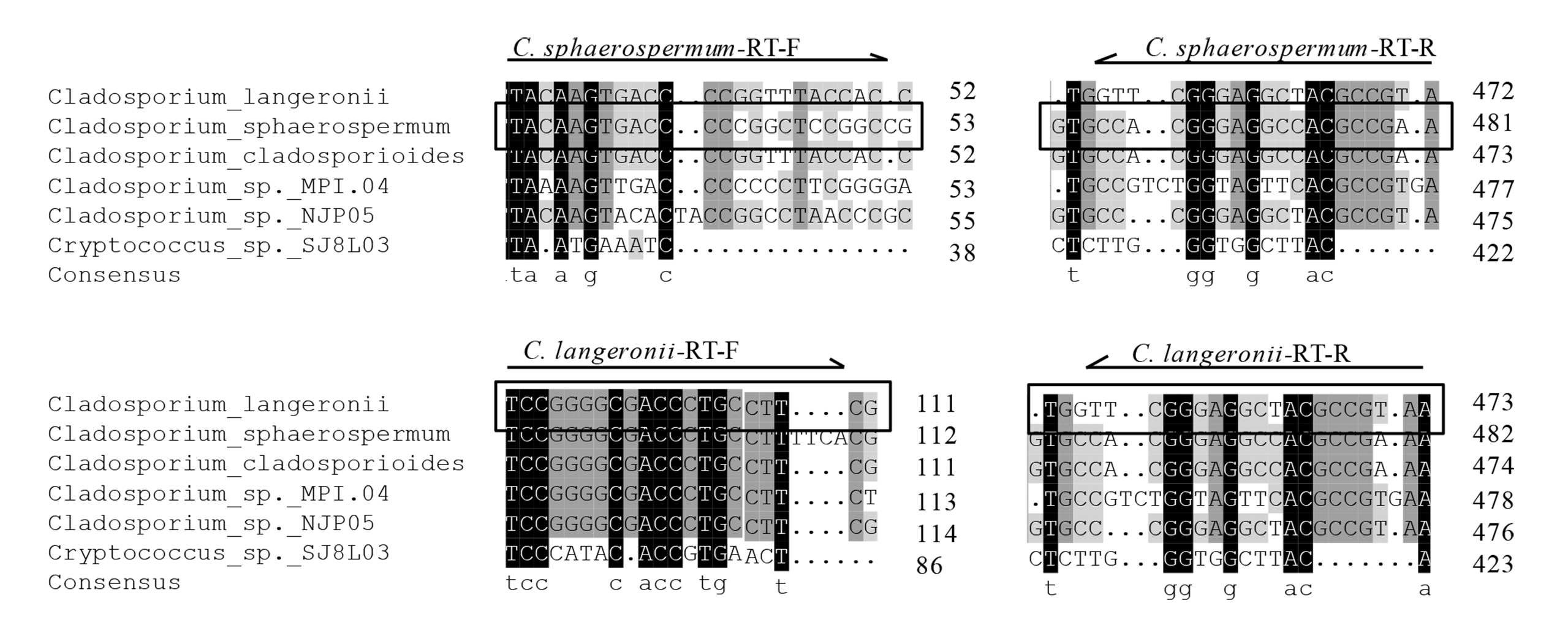


**Figure S6. The design of specific primers for *C. langeronii* and *C. sphaerospermum* in the ITS library sequences.**
